# An integrated practice system for learning programming in Python: design and evaluation

**DOI:** 10.1186/s41039-018-0085-9

**Published:** 2018-12-04

**Authors:** Peter Brusilovsky, Lauri Malmi, Roya Hosseini, Julio Guerra, Teemu Sirkiä, Kerttu Pollari-Malmi

**Affiliations:** 1 School of Computing and Information, University of Pittsburgh, 135 N. Bellefield Ave., Pittsburgh, 15260 PA USA; 2 Department of Computer Science, Aalto University, Espoo, Finland; 3 Instituto de Informatica, Universidad Austral de Chile, Valdiva, Chile

**Keywords:** Introductory programming education, CS1, Python, Multiple content types, Student motivation, Student performance, Classroom study

## Abstract

Over the past decades, computer science educators have developed a multitude of interactive learning resources to support learning in various computer science domains, especially in introductory programming. While such *smart content* items are known to be beneficial, they are frequently offered through different login-based systems, each with its own student identification for giving credits and collecting log data. As a consequence, using more than one kind of smart learning content is rarely possible, due to overhead for both teachers and students caused by adopting and using several systems in the context of a single course. In this paper, we present a general purpose architecture for integrating multiple kinds of smart content into a single system. As a proof of this approach, we have developed the Python Grids practice system for learning Python, which integrates four kinds of smart content running on different servers across two continents. The system has been used over a whole semester in a large-scale introductory programming course to provide voluntary practice content for over 600 students. In turn, the ability to offer four kinds of content within a single system enabled us to examine the impact of using a variety of smart learning content on students’ studying behavior and learning outcomes. The results show that the majority of students who used the system were engaged with all four types of content, instead of only engaging with one or two types. Moreover, accessing multiple types of content correlated with higher course performance, as compared to using only one type of content. In addition, weekly practice with the system during the course also correlated with better overall course performance, rather than using it mainly for preparing for the course final examination. We also explored students’ motivational profiles and found that students using the system had higher levels of motivation than those who did not use the system. We discuss the implications of these findings.

## Introduction

Over the last 15 years, researchers and practitioners in the field of computer science education put a lot of efforts to develop and explore advanced types of interactive learning content such as problem solving with automatic assessment (Hsiao et al. 2010; Ihantola and Karavirta 2011; Ihantola et al. 2010), interactive program visualization and algorithm animation (Shaffer et al. 2007; Sorva et al. 2013), intelligent tutoring systems (Kumar 2005; Mitrovic 2003), and adaptive electronic textbooks (Davidovic et al. 2003; Kavcic 2004; Weber and Brusilovsky 2001). Within the field, these types of content are frequently referred as “smart content” (Brusilovsky et al. 2014) due to its ability to offer individual guidance and feedback, complementing those traditionally provided by instructors. Classroom and lab studies with different kinds of smart content have demonstrated their benefits for student learning that resulted in better learning outcomes (Davidovic et al. 2003; Hosseini et al. 2016; Hsiao et al. 2010; Kavcic 2004; Kumar 2005; Mitrovic 2003). Yet, practical impact of these systems in the learning process has been much lower than expected (Naps et al. 2002). The main problem here is that the major fraction of smart content use was expected to come from non-mandatory (and not graded) student practice outside the classroom. In other words, the majority of smart content was developed to help students in mastering new topics after they were presented in class, assessing their knowledge, and getting ready to work on graded assignments and projects.

As it has been discovered in several studies, this non-mandatory practice context frequently failed to engage students to work with the smart content (such as program visualization) outside the classroom despite the efforts to convince them that this work will strongly benefit their knowledge (Naps et al. 2002). Only a fraction of students usually explored the non-mandatory content and many of those who tried dropped their practice after a few attempts. The engagement problem has been long recognized by researchers in the field of computer science education (CSE) who explored several approaches to better engage students to work with practice content. For example, for the case of algorithm animation, better retention and better learning results were obtained by engaging students in more interactive work with the visualization, instead of simply viewing an animation (Hundhausen et al. 2002; Naps et al. 2000). For the case of self-assessment problems, it has been found that personalized guidance and progress visualization could significantly increase the amount and regularity of student practice as well as learning outcomes (Brusilovsky and Sosnovsky 2005; Hsiao et al. 2010).

In this paper, we further investigate the problem of student work with non-mandatory smart content by introducing and exploring the idea of an *integrated practice system* that provides an organized unified access to multiple types of smart practice content. It is a plausible hypothesis that student learning and engagement can be increased if students can use *multiple* different types of smart content to learn. On the one hand, it would provide wider opportunities for students to explore and practice domain knowledge. On the other hand, since students often have preferred types of learning activities, the availability of multiple content types would provide more opportunities for students to focus on things that they consider to be beneficial for their learning.

The main problem in implementing this vision is that integrating multiple types of smart content into a single practice system is not technically trivial. The complexity of modern educational tools in CSE has reached the point when one academic research team has to focus on one kind of technology and one kind of educational content to stay on top of research and development trends. Given the high levels of interactivity and the advanced nature of modern learning content and tools, each team usually releases its own smart content and supporting tools in the form of a self-contained (and frequently login-based) system. We can cite dozens of modern computer science education (CSE) systems and environments, with each one usually offering one kind of smart content that support one aspect of CSE, for example, collections of animated algorithms (Shaffer et al. 2007), program visualizations (Sorva et al. 2013), practice systems offering one kind of programming problem (Hsiao et al. 2010; Ihantola and Karavirta 2011; Kumar 2005), systems for automatic assessment of programming assignments (Ihantola et al. 2010), and many others. As a result, the only option to use a wider variety of smart content in e-learning is a parallel use of several different login-based systems.

Unfortunately, using multiple separate learning environments in a single course is tiresome for instructors and confusing for students. Modern browsers, of course, provide some help here by remembering user names and passwords, as well as providing bookmarks to access content. However, even with such help, both students and teachers would still need to execute different content in separate tabs and/or switch between multiple bookmarks to access interactive content in a chapter. As a recent study shows (Sheshadri et al. 2018), students rarely work across platforms in a single session even if the use of each platform is required for the course. From students’ point of view, it is simply *not relevant* to recognize that one is using several different platforms, but they should be able to focus only on the content itself.

Another technical argument for the benefits of integration is data logging. Modern interactive learning contents allow to log student interaction data in great depth and apply this information to better understand the learning process and students’ progress. Yet, to make full use of this, collecting and analyzing data from multiple independent systems is considerably complex compared with integrated access and data logging. It is simply difficult to keep track of available exercises scattered throughout various places and to monitor learning progress over multiple systems.

The ACM ITiCSE working group on the use of smart content in computer science education (Brusilovsky et al. 2014) argued that the problems could be resolved by a software infrastructure that can seamlessly integrate multiple kinds of smart content while supporting centralized data collection about student progress. Following this approach, we present two contributions in this paper. Firstly, our team developed Python Grids, an open integrated practice system for learning Python. To build this system, we implemented an infrastructure for smart content integration that enabled us to use multiple kinds of smart content developed by different providers that reside on different servers worldwide. Secondly, the developed integrated system enabled us to explore an important open research question: *How does using multiple types of smart content benefit learning, as compared to using just one type of content?* To answer this question, we ran a classroom study in two different programming courses with over 600 enrolled students.

We believe that integrated practice systems, such as Python Grids, could be a valuable approach to present non-mandatory practice content to students in CSE and beyond. We first present the underlying architecture that enabled us to reuse multiple kinds of smart content. Secondly, we describe the details of our classroom study and present an extensive analysis of its results that focuses on how students used different types of practice content and how their work with content correlated with their own working practices and learning results.

## Previous work

As this paper presents two types of contributions, we discuss first relevant previous work related to technological developments of integrating multiple types of smart learning content. Thereafter, we discuss related empirical work of evaluating the use and impact of such content.

### Integrating smart content

The encapsulation of smart content into various systems has been long recognized as an obstacle both for re-using smart content and integrating multiple kinds of smart content within a single system. It has emerged as a new form of *interoperability* challenges in computing systems, and it is becoming one of the major issues for both academia and industry when developing new content and learning management systems. The interoperability of content means that the content is *reusable*, i.e., the same content can easily be used in multiple environments. Over the last 15 years, many researchers and practitioners have attempted to address the problem of smart content interoperability in the area of learning technology. In the middle of 2000, several infrastructures, such as KnowledgeTree (Brusilovsky 2004), MEDEA (Trella et al. 2005), and APeLS (Conlan and Wade 2004) were proposed by different research teams for integrating multiple kinds of smart content into a single system. These infrastructures also recognized and supported other aspects of working with smart content, such as data collection, learner modeling, and personalization. Later, an IMS LTI standard (IMS Global Learning Consortium 2010) emerged as an approach to support some aspects of smart content integration into LMS. LTI defined a standard way of launching online activities and sending the grade back to the learning management system. Nowadays, mainstream learning management systems, such as Moodle and Blackboard, support LTI-embedded content. While the LTI standard ignored the critical problem of learner data collection, it did help to develop more advanced infrastructures for smart content integration.

In a parallel research stream in the area of CSE research, several researchers have attempted to create learning environments that provide access to multiple kinds of smart content. Among the most notable of these efforts, we should mention Problets (Kumar 2005) and Ville (Rajala et al. 2007), which took opposite approaches to integrating diverse content. Problets offered a large collection of different assignments related to several different programming languages with loose integration: links to all of the problets were provided on a dedicated Web page (http://problets.org). While this system made problets highly reusable, each problet expected a separate login for data collection. In contrast, Ville (Rajala et al. 2007) integrated examples, pop-up questions, and program visualizations for different programming languages into an LMS style system with a single log-in, but it lacks advanced content offered by other systems and had no opportunities to re-use its own content outside of the system.

More recent CSE projects have explored some interoperability solutions to offer e-books, exercise systems, and massive open online courses (MOOCs) with one or two types of integrated smart content. Examples include the interactive textbook of CS principles (Ericson et al. 2015a), the OpenDSA textbook^1^ for data structures and algorithms (Fouh et al. 2014), the CloudCoder exercise system (Papancea et al. 2013), and the Aalto University MOOC on introductory programming in Scala^2^. Some of these systems used an existing interoperability standard, such as LTI (Fouh et al. 2014); in other cases, researchers developed simple interoperability protocols specifically to support their infrastructures (Brusilovsky 2004; Karavirta et al. 2013).

The Python Grids system presented in this paper could be considered as an integration of the two streams of research mentioned above. On the one hand, it could be considered as another attempt to assemble multiple kinds of smart content for the needs of computer science education. On the other hand, it is an exploration of an infrastructure for reusing smart content that follows the guidelines established by the ITiCSE working group (Brusilovsky et al. 2014). By using several interoperability protocols, it integrates several types of smart learning content that were independently developed by two research teams.

### Evaluating the impact of smart content

There is multitude of research carried out in developing and evaluating *single* type of smart learning content, such as program visualization (Sorva et al. 2013) and algorithm visualization (Shaffer et al. 2007), or automatic assessment of programming exercises (Ihantola et al. 2010). Based on the meta-study results of (Hundhausen et al. 2002), which highlight the importance of students’ engagement and activity with the content, Naps et al. developed an engagement taxonomy (Naps et al. 2002) to differentiate between various types of student activities with the content. The basic version covered six levels of engagement: *non-viewing* and *viewing* were the lowest levels where the student can just see something (or nothing) without any interaction. In the *responding* level, the system can pose questions to students to direct their attention to some aspects of the visualization, and in the *changing* level, students can explore the system behavior, e.g., by providing their own input data. In the highest levels, students can *create* novel content with the system or *present* and explain it to others. This taxonomy and some of its further developments, for example by Myller et al. (2009), have widely guided evaluation research, especially in algorithm visualization research but also elsewhere. However, these studies frequently focus on evaluating the impact of one type of content only, whereas in this research, we are interested in the impact of multiple types of smart learning content.

Another branch of research has focused on the development and evaluation of *ebooks*. Substantial part of research on ebooks focuses on analyzing how students and instructors use the standard ebook features, like table of contents, navigation options, bookmarks, etc. (Chong et al. 2009), as well as comparing students’ experiences with ebooks versus traditional printed books. This research falls out of scope of our work, because it does not consider interactive elements in the *book contents*. However, within the domain of computer science education, an increasing volume of research focuses on ebooks enriched by various kinds of smart content (Alvarado et al. 2012; Ericson et al. 2015a,, 2016; Fouh et al. 2014; Sirkiä and Sorva 2015). Among the types of smart content used in these ebooks are CodeLens program visualizations, ActiveCode widgets for editing and executing code, and Parson’s problems, i.e., constructing code by drag-and-dropping code lines into the correct order. These types of smart content are based on the technologies developed by Ihantola and Karavirta (2011); Guo (2013); Miller and Ranum (2012).

Alvarado et al. (2012) examined how students (*N* = 61) used the ebook widgets and how this correlated with their performance, measured as quiz results and midterm exam. The only significant findings were a medium level correlation between the usage of CodeLens tasks outside the classroom sessions and students’ performance in the mid-term exam or one quiz set in the ebook. Using ActiveCode widgets or watching videos did not have any significant correlation with performance measures. The authors speculated that regular use of CodeLens was the factor contributing to the higher learning gains. In a further study, Ericson et al. (2015a) analyzed how several hundreds of high school and college students had used various interactive components of the book. Here, the focus of the study was how much different components were used, and there was no comparison with the learning results.

Ericson et al. (2015b) explored usability issues of interactive components on three different ebook platforms (Runestone, Zyante, and CS Circles). This was followed by a small-scale evaluation study where 10 teachers used an ebook to learn programming. The results implied that in pre/post-test comparison, teachers who used more interactive components performed better. This was encouraging, but due to the small number of participants, the results were only tentative. In a further larger scale study (Ericson et al. 2016), they, however, analyzed only log data on how teachers used the material, as well as carried out a qualitative analysis of teachers’ feedback on using the book. Based on these results, they suggested several design principles for ebooks. No analysis of learning results was reported.

Other relevant studies of using multiple types of interactive content include the work of Fenwick et al. (2013). They built an interactive book for programming using Apple’s iBook Author application. The focus of analysis was more on the technical feasibility of the authoring process, and the collected data included only student feedback. Sirkiä and Sorva (2015) carried out a very detailed analysis of how students used program visualization widgets in a programming ebook. While the book included also other types of interactive elements, this study focused only on how animations were used.

In summary, our literature review suggests that there is a considerable need for further research on how students use online materials integrating multiple types of smart content and how this usage is related to their learning results. The study reported in this paper provides some more insight in this direction.

## The Python Grids system

The Python Grids is an integrated practice system that provides access to four types of interactive practice content for learning the Python language: annotated examples, animated examples, semantic code assessment problems, and code construction problems (known as Parson’s problems). We present below each of them separately. Thereafter, we explain how the students access the interactive content by using the *Mastery Grids interface* which provides a personalized access to the practice content and progress tracking (Loboda et al. 2014). Finally, we discuss the characteristics of the provided interaction types from the perspective of learning programming.

### Annotated examples

**Fig. 1:**
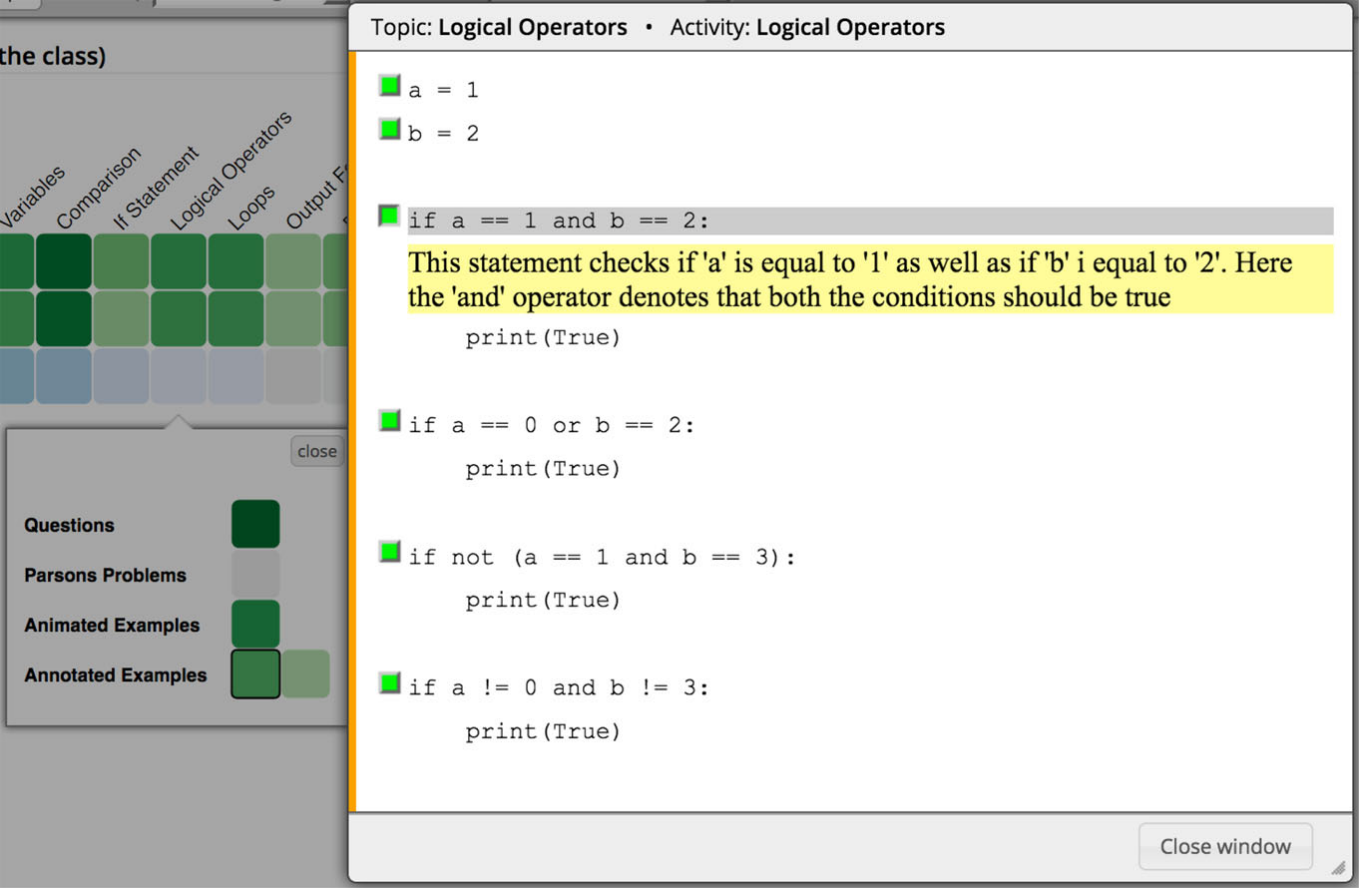
An instance of an annotated example in the Python Grids system. Here, the student has clicked on the third line and an explanation is shown below the line that demonstrates the result of executing this line in the example program

Annotated examples provide interactively explorable text explanations of code examples. Figure 1 illustrates an annotated example in the topic “Logical Operators.” A green bullet next to a line indicates that an explanation is available for that line. Once the student clicks on the line or the green bullet next to it, the explanation opens up below the line. Each explanation emphasizes important concepts in the line or the result of the line being executed.

Annotated examples are delivered by the WebEx system (Brusilovsky et al. 2009). All interactions of students with these examples are reported to the user modeling server by the WebEx system. The reported data includes information about each code line of an example that the student has viewed, along with the timestamp of those activities. We use this data in our analysis to evaluate the usage of examples and their impact on student performance.

### Animated examples

**Fig. 2:**
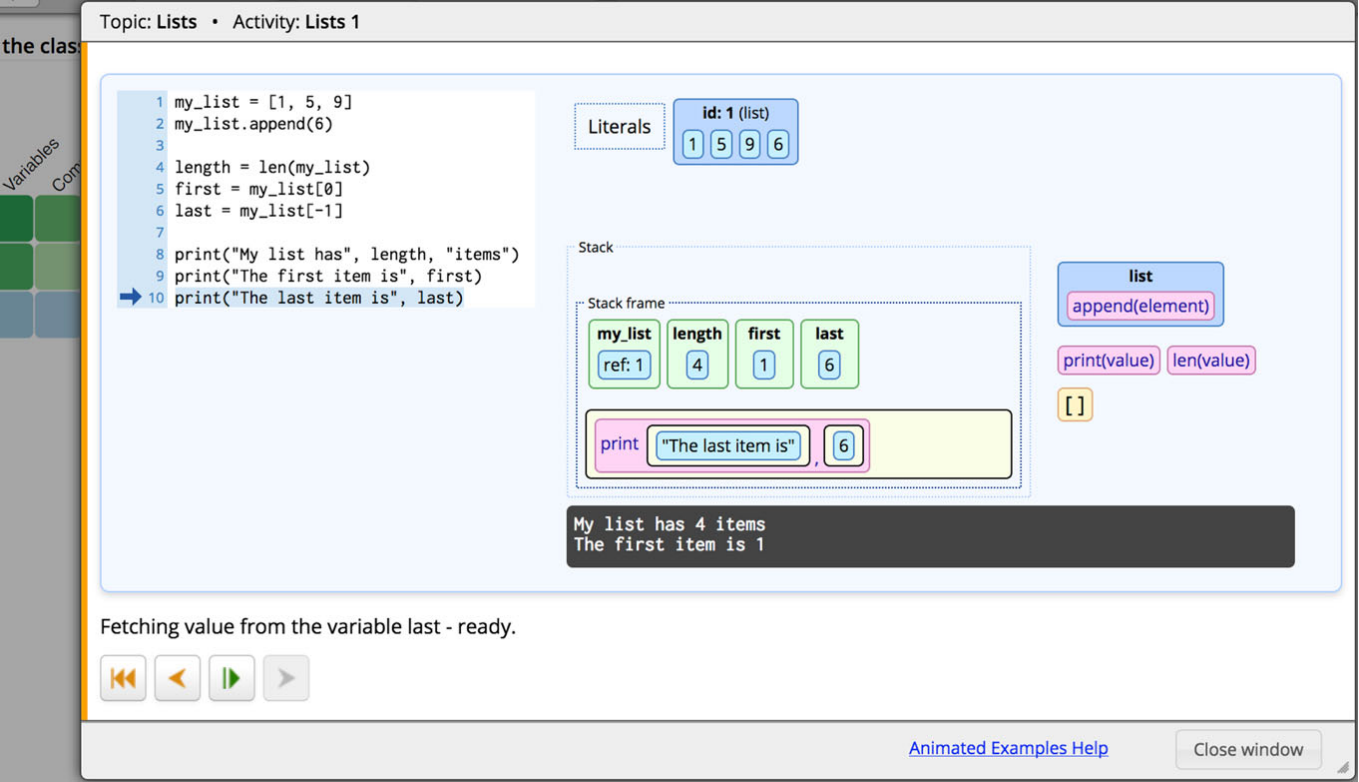
An animated example in the Python Grids system. The right panel shows the state of the stack frame and the output printed in the console when the program execution reaches the last line of the example

Animated examples (Fig. 2) provide an expression-level visualization of the code execution. The aim of these examples is to visually demonstrate how various programming constructs are executed by showing how each execution step changes the program state. These examples are implemented with the Jsvee library (Sirkiä 2018) and are delivered using the Acos content server (which will be explained in the next section).

### Python semantics problems

Semantic problems are parameterized exercises that test student comprehension of program execution by asking them about the output of the given program or the final value of a specific variable after the program is executed. These problems are generated by the QuizPET (Quizzes for Python Educational Testing) system, which is a re-design of QuizJET, an earlier Java-focused system (Hsiao et al. 2010). Since these exercises are parameterized, students can practice the same problem several times, each time with randomly selected values for the problem’s parameter.

**Fig. 3:**
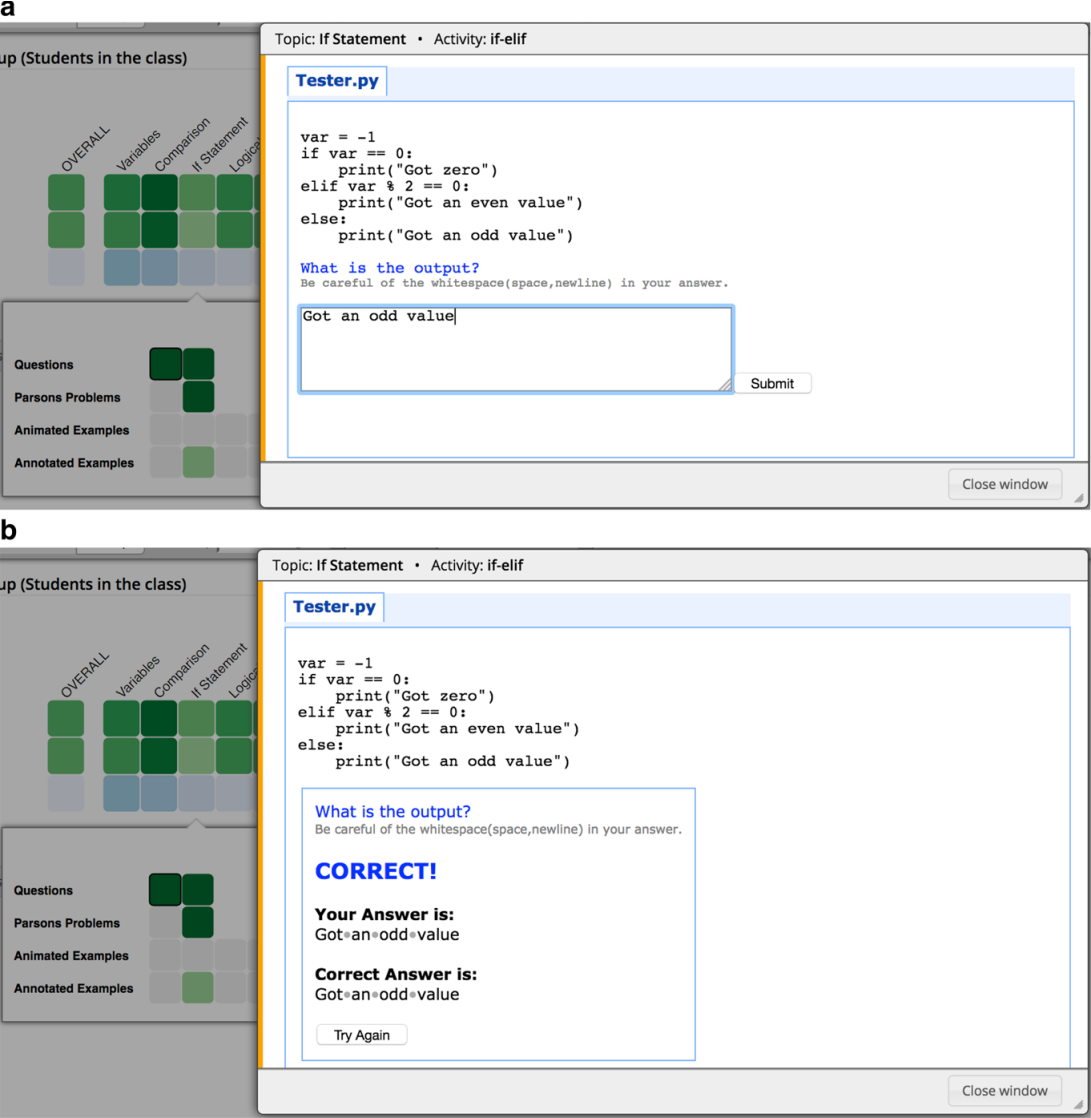
An instance of a QuizPET problem in the Python Grids system. **a** Before and **b** after student submits her answer for the problem

Figure 3a shows an instance of a semantic problem for the “If Statement” topic. The student writes her answer in the text box area below the problem. Once the student’s answer is submitted, QuizPET evaluates it and presents feedback to the student, along with the correct answer. Figure 3b shows the feedback presented to the student when the answer is evaluated as correct. The student can repeat the same problem with different parameter values by clicking on the “Try Again” button.

### Parson’s problems

Parson’s problems are code construction exercises in which students do not need to type code. This type of smart content was originally introduced by Parsons and Haden (2006) as *Parson’s Puzzles* where a limited number of code fragments is presented in a random order. Each fragment may contain one or more lines of code. To solve the puzzle, the student must construct the described program by putting the fragments in the correct order. This kind of exercises became quite popular and are now more frequently called Parson’s problems (Ihantola and Karavirta 2011; Ericson et al. 2015a).

**Fig. 4:**
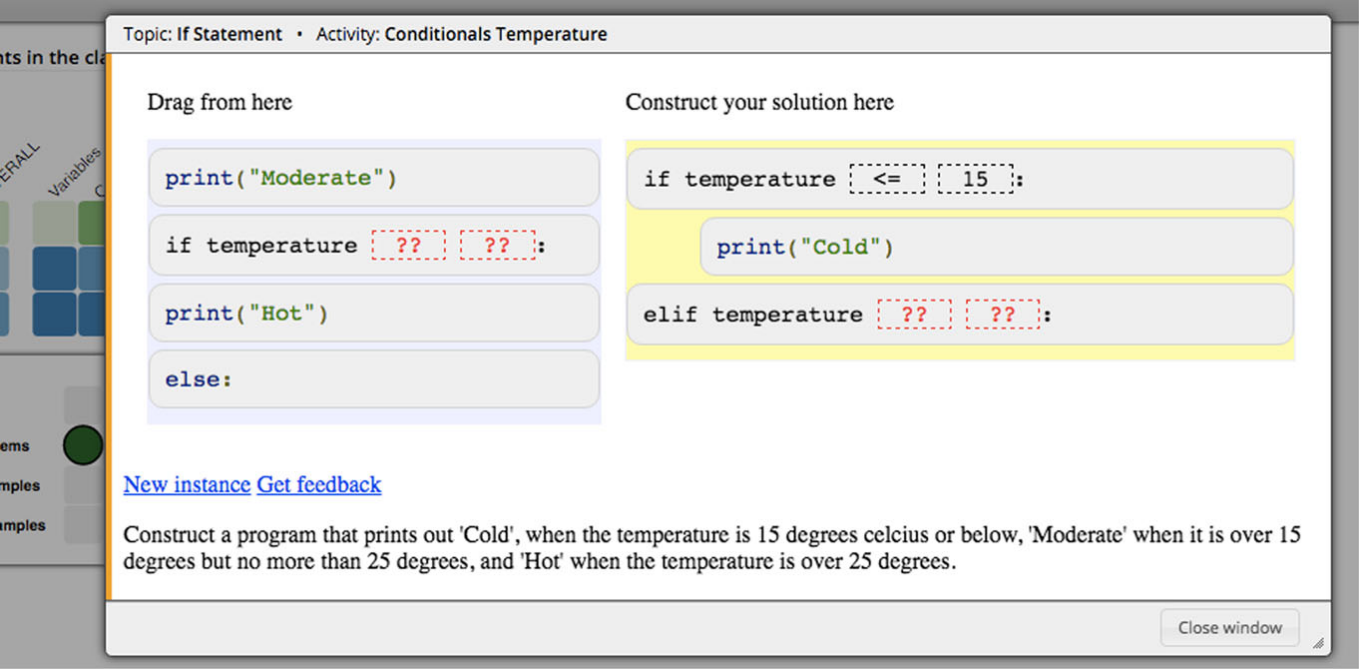
An instance of a Js-parson exercise in the Python Grids system. The student assembles the solution to the question (written at the bottom) in the right side

Figure 4 shows an instance of Parson’s problems in the Python Grids system. At the bottom, there is a description of the code that should be constructed. The exercise is solved by dragging and dropping the required code fragments from left to right in the correct order.

Parson’s problems in Python Grids are implemented with a JavaScript Js-parsons library^3^ implemented by Ihantola and Karavirta (2011) and delivered by the Acos server (Sirkiä and Haaranen 2017). On top of the original implementation, Js-parsons requires students to indent their code correctly. Therefore, the fragments must not only be in the correct order, but must also correctly indented. This is an important aspect in Python because the indentation has a semantical meaning. Instead of solely giving the feedback based on the positions of the fragments, Js-parsons exercises have unit tests that validate the correct runtime behavior.

In addition, the Js-parsons library supports distractors, i.e., all the given code fragments may not be necessary for the solution. Distractors make the unwanted trial-and-error approach more difficult because the extraneous fragments increase the possible number of combinations and force the student to think more. When the task is completed, the distractors remain in the left side and only the correct fragments are part of the solution.

The fragments may also contain toggleable elements which are shown as gaps. For these fragments, the student must select, for example, the correct operator or variable name to fill the gap (see the segmented squares with question marks “??” in Fig. 4).

### The Mastery Grids interface for personalized content access

**Fig. 5:**
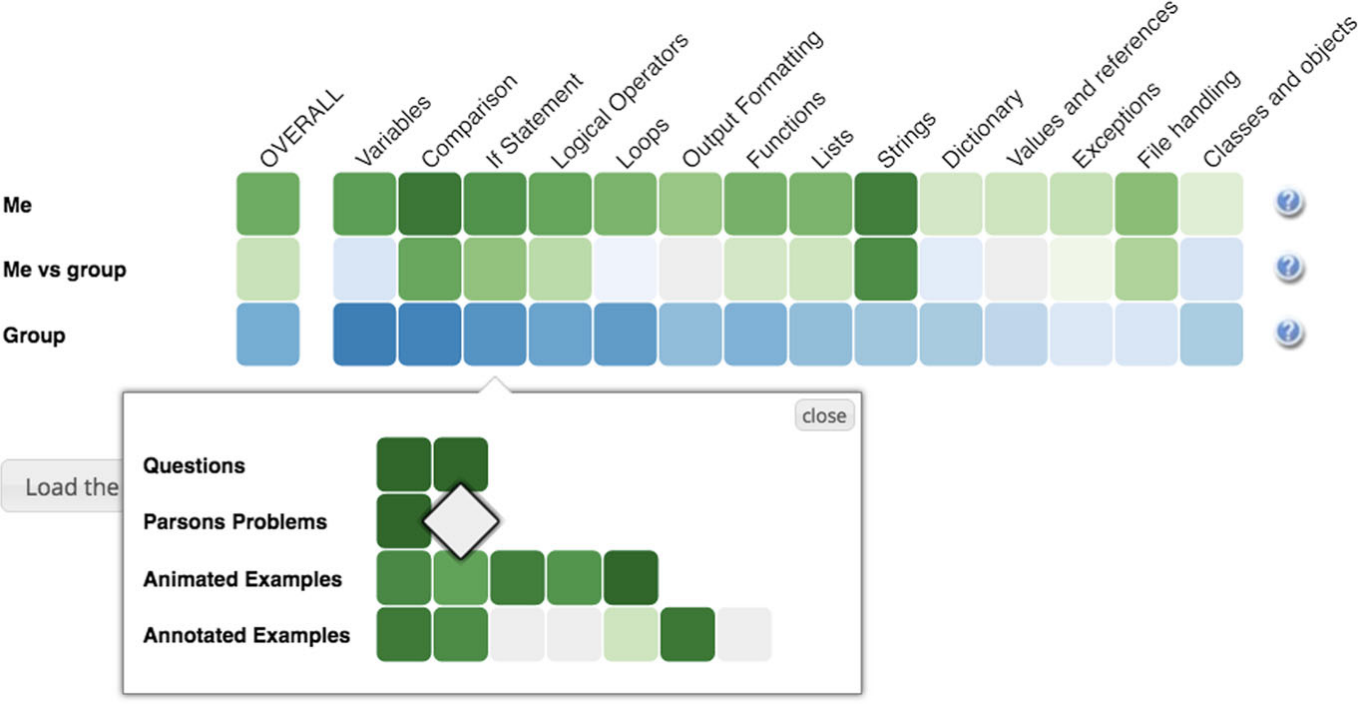
The Python Grids system

Two important values of an integrated practice system are the ability to access several types of content through the same interface and the ability to offer a unified progress tracking. In Python Grids, a unified access and progress tracking is provided by Mastery Grids, a personalized content access interface with *open social student modeling* (Loboda et al. 2014). The Mastery Grids interface (Fig. 5) supports students by visualizing their topic-by-topic progress and comparing it with other students in the class. It visualizes a course as a grid of *topic cells*, which are ordered as they are covered in the course and are colored according to the level of student progress within each topic. In the current version of Python Grids, the progress of an activity or a topic is computed as the proportion of completed activities, i.e., the color density of an example activity indicates the fraction of explored annotations or animations. The color density of a problem cell indicates whether it has been solved correctly or not. The color of the topic indicates the cumulative amount of work with all activities in the topic. The darkest color indicating a fully completed topic. Each time the student completed an activity, its topic cell darkens proportionally. In Fig. 5, the first row represents the progress of the current student with a green color of a different intensity. The third row represents the progress of a reference group (for example, the rest of the class) with a blue color of a different intensity. In both cases, a darker color indicates a higher level of progress within a topic. The middle *comparison* row visualizes the difference between the student and the group, namely, cells here will be green if the student has a higher level of progress than the group, and blue if she is behind the group. The color intensity shows the magnitude of differences. If student progress is equal to group progress, the topic cell is shown in neutral gray.

By clicking on a topic, the student can open the *content collection* for the topic, which is shown as a grid of *activity cells* organized in rows representing different types of content. In Fig. 5, the student has clicked on the topic *If Statement* and opened a content collection with 16 content items in it: two semantic problems, two Parson’s problems, five animated examples, and seven annotated examples. Clicking in an *activity cell* opens the corresponding learning activity in an overlaid window, as shown in Figs. 1, 2, 3 and 4. The following subsections provide more details about each of the four types of activities used in Python Grids. Once the student has completed the activity, the activity window is closed and student progress for the activity and its topic is immediately updated in the Mastery Grids interface.

### Characteristics and pedagogy of the provided content

The types of content support students’ learning both in *code comprehension* and *code construction*. In the annotated examples, students can choose the code lines where they feel they need an explanation and dismiss explanation provided for other lines. Explanations describe the line of code and its role in the whole example, and in this way support knowledge towards both code comprehension and construction. Animated examples target code comprehension only; they provide dynamic information instead of static explanation. The student can browse code execution step by step forwards and backwards and visually explore how the program state changes and expressions are evaluated and in which order code lines are executed. The third type, semantic code assessment problems, provides a different type of activity, where the student has to provide answers to questions by tracing code execution. This supports code comprehension. The final type, Parson’s problems, focuses on training code construction skills. The student has to build a working program by dragging and dropping given code lines into a correct order and choosing appropriate operators and operands in expressions. Naturally, these problems also train code comprehension.

Our basic pedagogical idea combines these resources as follows. When the student starts to work with a new concept, he/she can first obtain the general idea of the concept by reading the annotated examples and opening the explanations when needed. Thereafter, the student can enhance his/her understanding by viewing how the concept concretely works in animated examples. Next, the student can test his/her understanding of the concept with the semantic code assessment problems and correct possible misconceptions. Finally, solving Parson’s problems is the first step to extend the skills from code comprehension to code construction. Ideally, the student would start solving the compulsory programming exercises only after finishing all four types of voluntary practice content.

From another perspective, both types of examples present visual and descriptive explanations, while problems offer self-assessment. In Table 1, we summarize this categorization in a 2 × 2 table with two dimensions: interaction (exploratory or assessment) and support (comprehension or construction).

**Table 1:** Characteristics of the different types of content in Python Grids

	Comprehension	Construction
Explanatory	Animated examples	Annotated examples
	Annotated examples	
Assessment	Semantic code assessments problems	Parson’s problems

Finally, all these types of content also provide support for different levels of student engagement in terms of the engagement taxonomy presented in Naps et al. (2002). The first two types (annotated and animated examples) work in *viewing level*, the third one (semantic code assessment problems) in *responding level*, and the final one (Parson’s problems) in *constructing level*.

## Integrating multiple kinds of smart content: architecture and protocols

This section explains the server side of Python Grids: its underlying architecture that makes the integration of several types of smart content possible. As explained in the “Introduction” section, Python Grids is an attempt to implement the vision of the ACM ITiCSE working group on the use of smart content in computer science education (Brusilovsky et al. 2014). It brings together several types of smart learning content that are independent of the host system, fully re-usable, and hosted by different physical servers that are, in fact, located in different countries. We already mentioned that the animated examples and Parson’s problems are hosted on the Acos server^4^ located in Espoo, Finland. Acos 2017 is designed to provide the same smart learning content to multiple learning management systems that use different *interoperability protocols* and can host multiple types of content. Semantic problems and annotated examples are served by specialized QuizPET and WebEx content servers, respectively, that are located in Pittsburgh, USA. In this context, the Mastery Grids interface works as an *aggregator* that contains links to the content that are hosted in different content servers and transparently delivers the selected content to the students. The students might not be aware of which external system actually provides each type of content—what they see is a holistic Python Grids system with the Mastery Grids interface with diverse learning content.

The ability to provide such transparent access to multiple kinds of reusable content while supporting data collection and personalization is supported by the Python Grids infrastructure, which is a considerable extension of the original KnowledgeTree framework (Brusilovsky 2004). Python Grids introduces communications protocols based on JSON, the ability of integrated monitoring of knowledge and progress (progress on the content), and independence of the learner model (user model) server.

**Fig. 6:**
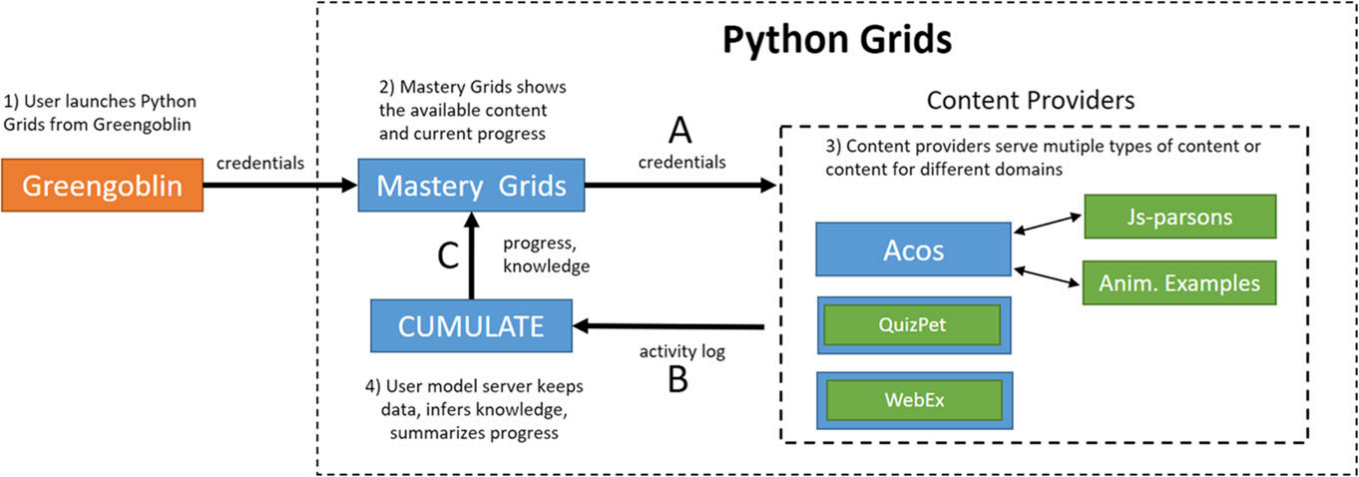
An overview of the different components in the Python Grids system and the communication that occurs between them. The arrows indicate the direction of the communication. For example, Mastery Grids never communicates with Greengoblin, but animated examples have two-way communication with Acos. The explanations give an overview of how information is transferred when a student visits Python Grids and uses the content provided by an external server. The orange box represents the learning management system in Finland which gives access to Python Grids, blue boxes are servers located in Finland and the USA, and green boxes represent different content types. Note that QuizPET and WebEx content types are shown inside a small blue box, which indicates that there is a dedicated server that hosts only this type of content

The infrastructure includes several types of components that inter-operate by using standardized communication protocols (Fig. 6). The main types of components are smart content servers as mentioned above, learning portals such as the Python Grids front-end with the Mastery Grids interface, and student modeling servers, such as the CUMULATE (Yudelson et al. 2007).

Three communication protocols support the smooth co-operation between the independent components within the Python Grids system. The first *content invocation* protocol (the arrow labeled with letter A in the Fig. 6) defines how a learning content item could be invoked from a specific server by a portal (i.e., from the Mastery Grids interface). The protocol is implemented as an HTTP GET request to the content provider, which identifies the requested activity and also passes the user’s credentials: user identifier, session identifier, and group (class) identifier. The content is loaded into an *iframe*, and there is no further direct communication between the content interface and the Mastery Grids interface. This first protocol imposes a requirement on the content provider: single content items should load independently into an iframe through a unique URL.

The second *event report* protocol (the letter B in Fig. 6), also known as the CUMULATE protocol^5^, defines how learning data is reported and logged to the student modeling server. Interactive content generates *learning events* based on user actions. For example, each time a student moves to the new line in the Jsvee animations, Jsvee will send an event to Acos, which will deliver the event to the student modeling server CUMULATE using the learner modeling protocol. QuizPet problems and WebEx examples send learning data as a flow of events directly to the student modeling server. CUMULATE uses the flow of learning events to calculate the knowledge progress on several levels that range from the whole topic to an individual content item. Since each type of content may require a different approach to compute the progress that a student has made on it, the student model needs to know how learning events can be processed to estimate knowledge progress. A set of services in the CUMULATE user model has been developed to provide such computations for all four types of content in the Python Grids system.

The flow or educational events sent to CUMULATE contain only as much information as is necessary to keep track of learning progress. In addition to this “integrated” log, different content servers might keep their own detailed log data that can be used for specialized data analysis. For example, Jsvee animations track how many seconds students spend at each animation step, how students proceed with the animation, and other pertinent information. In addition to sending learning events to the student modeling server, Acos provides a mechanism to store arbitrary logging events to the server instead of sending them forward. In this case, the log files can be extracted from the Acos server when the data is needed. The second protocol imposes the reporting requirement over the content servers: they must comply with the protocol to log learning event information to the student modeling server.

The third *knowledge query* communication protocol (the letter C in Fig. 6) defines how portals or content servers can request information about student and group knowledge progress from the student modeling server. This communication channel is important to support personalization, learning analytics, and knowledge visualization. In the context of Python Grids, this information is used to present the comparative knowledge visualization shown in Fig. 5. To obtain this information, the aggregate server, which provides the server-side intelligence for the Python Grids, sends a knowledge query to the student modeling server CUMULATE.

Note that although the choice or KnowledgeTree framework (Brusilovsky 2004) as a basis for Python Grids implementation caused us to use the original KnowledgeTree protocols, by their nature, these protocols are similar to protocols used in other distributed e-learning systems. For example, CUMULATE protocols for reporting student performance and requesting student knowledge are similar to the protocols used by other user modeling servers such as PERSONIS (Kay et al. 2002) or LDAP-UMS (Kobsa and Fink 2006). An attempt to standardize the performance reporting protocol has been recently made by ADL in its xAPI standard (AdvancedDistributedLearningLab), and a popular standard for single sign-on content invocation protocol has been suggested by IMS (IMS Global Learning Consortium 2010).

With this data flow design, the components have their own tasks that make them highly re-usable. For example, the main task for smart content providers is to deliver smart content activities and maintain student interactions with them. The content does not have to worry about authentication, storing grades, or logging interaction data because there is a predefined interface of how to communicate with the other parts of the system. As a result, the architecture is fully open. New content servers could be easily added to offer other kinds of smart content just by implementing content invocation, and event reporting protocols (Acos server (Sirkiä and Haaranen 2017) provides an example of a new content server integration). Once a content server is connected to the architecture, all content available on this server are easily connected to a portal. Specifically, Mastery Grids interface offers a authoring tool for instructors, which supports the process of topic-level course structuring and drag-and-drop content assignment to specific topics (Chau et al. 2017). Moreover, different portals could be designed to maintain different types of interfaces with students, such as a more traditional “folder” interface of learning management systems, or electronic books such as Open DSA (Fouh et al. 2014).

The presence of standard communication protocols also simplifies the integration of Python Grids with other learning systems. For example, in the context of the study presented below, Aalto University students accessed the Python Grids system from Greengoblin, a local learning management system, which is shown as orange box in Fig. 6. The link to the Python Grids non-mandatory practice content was placed in the bottom of the front page of the Greengoblin interface. The integration between the Greengoblin system and Python Grids was implemented through a simple GET request, which authenticates the student to Python Grids and loads it in a separate window.

## A study of an integrated practice system

The implementation of a multi-content practice system supported by the integration architecture introduced above enabled us to perform a unique large-scale study that examined how students use multiple kinds of smart content in a practice mode. In the context of this paper, the study could be considered as a “proof” of our concept of integrated practice system. However, it also provides an important contribution to the topic by offering an empirical evidence on how different kinds of smart content offered in parallel are used in a semester-long course and what is the relation of different usage patterns to learning results.

Our main research question here is: *How does using multiple types of smart content benefit learning, as compared to using just one type of content?* To investigate this closer, we looked at the following subquestions:

- *RQ1. How do students use practice content offered by the Python Grids?*
- *RQ2. How is their content usage profile (how much and what type of content they use, and when) connected to their progress and learning results in the course?*

Based on previous experience of giving the course, we anticipated that the large student cohort in a service course includes students with quite different motivation towards programming. Moreover, the different motivations of the students may have a specially strong impact in the engagement with Python Grids because this system is offered as a voluntary and complementary system. Therefore, we collected data from students’ motivational profiles to better interpret the results to respond to the following subquestion:

- *RQ3. How is students’ motivational profile related to their learning results and usage profile?*

This section presents the details of the study while the next section examines the most important results. We emphasize here that while these research questions could also be explored without integrating smart content together, our setting provides some unique advantages. Firstly, from students’ perspective, an integrated system is easier to use, when all resources can be accessed in a uniform way, without logging into several systems separately. On the other hand, without student identity information, gained through the login access, this study would be impossible to carry out. Secondly, integrating the content allows easy logging of all data and activities compared with collecting data from several separate systems.

### Designing the study

The study was carried out in fall 2015 at Aalto University in Finland. Python Grids was offered as a practice system to students who were enrolled in the introductory programming course, named “CSE-A1111 Basic Course in Programming Y1.” This course is a typical CS1 service course for students who are not Computer Science majors. Most of the students who take this course are not even Computer Science minors and will only take one to two programming courses in their university career. This basic course is compulsory for the School of Engineering and the School of Electrical Engineering. Normally, students will take this course during the first fall semester of their bachelor’s degree studies. The programming language used in the course is Python, and the course focuses on procedural programming. The contents of the course include all basic structures like variables, selection statements and loops, functions, parameters, lists, and handling text files. Only the basics of object-oriented programming are covered in the course.

The course grade is based on mandatory exercises, voluntary practice content, and a compulsory exam. The exam grade is weighted at 50% of the final grade, and the mandatory exercises and practice content contribute to the other 50%. Most of the mandatory exercises are small programming tasks (from 1 to 100 lines of program code), but 15 animated code examples and 11 Parson’s problems are also included. Over 92% of the maximum points of the mandatory exercises are given for solving the programming tasks, and less than 8% for watching animated code examples and solving Parson’s problems. The mandatory exercises are split into nine rounds. To pass the course, the student has to obtain about 50–60% of the maximum points of each round, except the last one that contains problems in object-oriented programming. This implies that passing the course does not require that students should correctly solve all exercises, but that they have some freedom in choosing their assignments if they do not aim at getting the highest grades. Regardless of this, we call all these exercises mandatory exercises to contrast them with practice content, which are additional voluntary learning resources.

All mandatory exercises were accessed through the Greengoblin course management system, which is shown in Fig. 6. All four types of Python practice content reviewed in the “The Python Grids system” section were accessed through the Python Grids system, using the interface shown in Fig. 5. As mentioned above, Python Grids was accessible from Greengoblin through a direct link. In total, the learning materials in Python Grids included 37 semantic problems, 32 Parson’s problems, 39 animated code examples, and 59 annotated code examples, which were all organized into 14 topics. If the student solved 15 voluntary problems or examples, he/she received a 3% bonus to his/her exercise points. The purpose of the bonus was to encourage the students to practice with the Python Grids system.

Our study followed a pre/post-test experimental design to examine the effect of the practice system on student learning. Students were asked to take a pretest and a post-test at the beginning and end of the course, respectively. Each test contained 10 Python problems, covering the following topics: variables, arithmetic expressions and assignment, if statement, loops with while and for statements, lists and indexing, functions and parameters, strings, reading a text file, handling exceptions, and basics of classes, objects, and methods. The student was either asked to give the final value of a certain variable in the end of the given code fragment or predict the output of the code fragment. Post-test problems were isomorphic to the pretest.

Additionally, students completed a motivational questionnaire at the beginning and at the end of the term. The motivational questionnaire contains two instruments: a learning activation questionnaire and an achievement-goal orientation questionnaire. The learning activation questionnaire was developed to measure motivation in learning sciences and included four motivational factors: fascination, competency beliefs, values, and scientific sense-making (Moore et al. 2011). From this questionnaire, we kept a core set of questions for the factors of fascination (four questions), competency beliefs (five questions), and values (five questions). We did not include questions about scientific sense-making because this factor corresponds to domain-specific skills. Since the original questions were designed for the subject of *science*, we modified these questions by maintaining the phrasing but changing the subject to *computer programming*. Items of in *fascination* factor measure the extent to which the student likes programming (“In general, I find programming…” with options “very boring,” “boring,” “interesting,” “very interesting”). *Competency beliefs* questions ask students if they think they can deal positively with the subject (“I can figure out how to finish a programming class project at home” with answers on a 5-point scale from “I’m sure I CAN’T do it” to “I’m sure I CAN do it”). *Values* questions measure to what extent students think programming will be important to their lives and professional development (“I think programming will be useful for me in the future” with options “NO,” “no,” “yes,” and “YES”).

The achievement-goal questionnaire is a 12-question survey that measures Goal-Orientation, a fundamental motivational factor in self-regulated learning experiences (Elliot and Murayama 2008). Goal-Orientation is comprised of four factors that are not exclusive: the *mastery approach* orientation is related to the motivation of mastering the learning content (“My goals is to learn as much as possible”); *mastery avoidance* is related to the avoidance of failing to learn (“My aim is to avoid learning less than I possibly could”); *performance approach* relates to motivation to perform, score, or do better than others (“My goal is to perform better than the other students”); and *performance avoidance* is being motivated to avoid to fail, score under the minimum, or do worse than others (“My aim is to avoid doing worse than others”). Each factor has three questions. Questions are measured on a 7-point scale with extremes labeled as “Not at all true of me” and “Very true of me” and a middle point “Unsure.” While the focus of this paper is not the study of motivational factors, these measures are important to help explain usage patterns and engagement with learning content.

### Collected data

The study included data from 675 students who participated in the mandatory exercises and obtained a non-zero amount of points in at least one of the nine exercise rounds; 563 of them received enough points from the mandatory exercises to pass the course, and 552 students passed both the exercises and the exam. Out of those students, 424 took both the pretest and post-test. There were 25 students whose performance in the post-test was lower than the pretest (negative learning gain). Following a common argument that negative learning does not occur during an active learning process, we hypothesized that these students may not have taken the post-test seriously or had already very high pretest which gives little room to show learning in the post-test given the limitations of the instrument (small set of questions). Indeed, from these 25 discarded cases, 7 were students with very high pretest (above 0.75), and 15 students with lower pretest were not active in Python Grids. Only 3 students were active in the system and have low pretest. At the end, following similar studies with less reliable assessment (Colt et al. 2011), we decided to remove 25 negative learning gain cases, i.e., for analyses that include pretest and post-test (or learning gain), we only consider 399 students.

The collected data on compulsory exercises included not only the points that students obtained from the exercises, but also the hours they spent while coding the compulsory exercises. The sum of median time the students reported for mandatory exercises was 41.4 h. This number was obtained by calculating the median separately for each problem (accounting for only those students who submitted the solution to the problem) and then summed the medians together. For novice students (i.e., those who had no programming experience at all at the beginning of the course), the sum of the medians increased to 46.6 h. In our data, we found only one extreme case, where the self-reported time was too extreme (2196 h). We treated that number as an outlier and thus excluded it from any analysis related to exercise efficiency.

Regarding usage of Python Grids, a total of 485 students logged into the system at least once. Among those, 336 (69.3%) attempted at least one content type and 298 (61.4%) were, by our definition, active—they either solved 5 problems or worked with examples for at least 5 min. We discarded *inactive* (or not active enough) students in this work, because we were interested in studying the effects of the use of multiple types of content within a system that was offered as voluntary and complementary practice resource. As such, we understand that many students may have not been engaged with the system because of many reasons other than the system itself, such as lack of time to work with voluntary content, poor motivation to learn programming, or self-confidence that there is no need for additional training.

We summarized the collected data on the usage of Python Grids using the variables listed in Table 2. We measured usage of practice contents (semantic and Parson problems) by counting the number of problems that students solved correctly. Both problem types had a distribution close to normal for the number of problems solved, with the average ratio of solved problems being *M*=0.50 and SE=0.01 for semantic problems and *M*=0.59 and SE=0.01 for Parson problems.

**Table 2:** System usage variables in Python Grids

Variable name	Description
Semantic problems	Number of semantic problems that student solved
Parson problems	Number of Parson problems that student solved
Annotated examples	Number of annotated examples that student viewed
Annotated examples lines	Number of lines that student checked while viewing annotated examples
Annotated examples duration	Amount of time (in seconds) student spent on annotated examples
Animated examples	Number of animated examples that student viewed
Animated examples lines	Number of lines that student checked while viewing animated examples
Animated examples duration	Amount of time (in seconds) student spent on animated examples

For annotated and animated examples, the amount of work with examples could be counted as the number of accesses to examples and the “depth” of access, which is critical for “instructional” content. To see whether these two aspects should be treated separately, we looked at the correlation between the number of examples viewed (access to examples) and the number of example lines viewed (depth of access), and we found that the correlation was very high for both the annotated examples and animated examples (*ρ*=0.98, *p*<0.0001). Additionally, to make sure that students worked with examples and did not just quickly click through the example lines without reading the annotations or reflecting on what the example visualized, we looked at the correlation between the number of examples viewed and the amount of time students spent on working with the examples. Again, we found the correlation to be very high for each example type (annotated examples: *ρ*=0.82, *p*<0.0001; animated examples: *ρ*=0.92, *p*<0.0001). This suggests that when accessing examples, students worked with them and did not just click through them. As a result, one of the measures of the work with examples is sufficient for later analysis; we selected the number of accesses.

The summary statistics of all system usage variables are shown in Table 3 for the active students. On average, students worked with more than 10 learning materials from each smart content type. The last row in the table shows the total usage of practice content regardless of content type; and it shows that, on average, students practiced with about 59 learning materials (i.e., about 35% of all available learning materials in the system counting semantic problems, Parson’s problems, animated and annotated examples). It is noteworthy that this number is about four times more than the number of problems that students had to solve to get the 3% bonus points. Thus, this data suggests that there were groups of students who were truly engaged by the system and who practiced with the system beyond any extra-credit incentives.

**Table 3:** Summary statistics for usage of smart content by active students who either solved five problems or worked with examples at least for 5 min (*N* = 298)

	Min	Median	Mean	SD	Max
Semantic problems	0	9	12.25	9.57	37
Parson problems	0	7	11.67	9.64	31
Annotated examples	0	16	20.5	16.57	57
Annotated examples lines	0	101	134.65	116.44	462
Annotated examples duration	0	222.51	487.14	891.68	10,692.87
Animated examples	0	11	14.94	12.13	39
Animated examples lines	0	62	112.64	115.44	478
Animated examples duration	0	217.37	540.3	828.87	7391.21
All types of content	5	44	59.35	43.87	160

## Data analysis

As stated in our research questions in the “A study of an integrated practice system” section, the goal of our data analysis was to understand how students use practice content offered by the Python Grids, how their content usage profile is connected to their progress and learning results in the course, and how their motivational profile can be used to interpret the results. The following paragraph provides a summary of our data analysis connecting it with the research questions. In addition, Table 4 provides an overview of the data analysis, illustrating the method that we used to answer each of our research questions and pointing to sections where the corresponding analysis is provided.

**Table 4:** Overview of the data analysis

Research question	Goal	Method	Section
RQ1	Understanding how students use practice content	Session-based analysis	“Were students using multiple types of smart content?” section
		Cluster-based analysis	“The value of practicing with multiple types of smart content” section
RQ2	Understanding how usage profile is connected to students’ progress and learning results	Regression-based analysis	“Were all types of smart content helpful?” section
		Regression-based analysis for comparison of learning outcomes between clusters that represent usage profile	“The value of practicing with multiple types of smart content” section
RQ3	Understanding how students’ motivational profile is related to their usage profile and learning results	ANOVA analysis for comparison of motivational profile between clusters that represent usage profile	“The impact of student motivation on using multiple types of smart content” section

After introducing the performance measures in the “Performance measures” section, the “Were all types of smart content helpful?” section examines connections between the use of each type of content and performance. The goal is to search for evidence that all types of learning material are valuable before deepening into more complicated analyses. The “Were students using multiple types of smart content?” section targets the research question *How do students use practice content offered by the Python Grids?* by looking at to which extent students did use multiple types of content. The “The value of practicing with multiple types of smart content” section addresses the research question *How is their content usage profile (how much and what type of content they use, and when) connected to their progress and learning results in the course?* using two cluster analyses grouping students by their pattern of usage of multiple content and by their pattern of usage of the practice system towards the term. The “The Impact of Student Motivation on Using Multiple Types of Smart Content” section explores the relation between system usage and learning motivation addressing the research question *How is students’ motivational profile related to their learning results and usage profile?* In these analyses, we relate motivational variables with the different clusters discovered in the “The value of practicing with multiple types of smart content” section. Finally, the “Profiles of students who did not use the system” section analyses data about the students who did not use the practice system at all. We think this section is important in the context of our classroom study where students made their own decisions whether to use our system and to which extent.

### Performance measures

The performance measures that we used in our analysis included (1) *learning gain*, which is defined as the ratio of the actual gain (post-test score minus pretest score) to the maximum possible gain (maximum achievable post-test score minus pretest score); (2) *exercise points* that a student received from nine rounds of mandatory exercises; (3) *exercise efficiency*; and (4) *exam points*, which a student received from the compulsory final exam.

Exercise efficiency is a measure that connects the total points gained by a student in the mandatory exercises and the amount of time that the student spent on coding exercises. The exercise points represent a student’s performance on mandatory exercises, and the time that the student spent on the coding part of these exercises approximates her/his mental effort. The mental effort is defined as the total amount of controlled cognitive processing in which a subject is engaged. Measures of mental effort can provide information on the cognitive costs of learning, performance, or both. We calculated the efficiency based on the approach of Paas and Van Merriënboer (1993). More specifically, we transformed both mandatory exercise points and the time students spent on mandatory coding exercises to standardized *z* scores (represented by *P*_*z*_ and *M*_*z*_, respectively) and then combined the *z* scores in the following manner to obtain the exercise efficiency score:

1 $$ E=\frac{P_{z}-M_{z}}{\sqrt{2}}  $$

The sign of the efficiency score depends on the relation between the total points that a student obtained from mandatory exercises (i.e., student’s performance in mandatory exercises) and the time that a student spent on the coding part of those exercises (i.e., invested mental effort on mandatory coding exercises). If *P*_*z*_−*M*_*z*_≥0, then *E* is non-negative; otherwise, *E* is negative. A zero/positive/negative efficiency represents the same/higher/lower performance on mandatory exercises in relation to invested time on the mandatory coding exercises; thus, the greater the value of the exercise efficiency, the more efficient a student was on mandatory exercises.

The summary statistics for the four performance measures are shown in Table 5. All values are drawn from student data.

**Table 5:** Summary statistics for performance measures

	Min	Median	Mean	SD	Max
Learning gain (*N*=399)	0	0.56	0.52	0.29	1
Exercise points (*N*=640)	270	5360	4842.55	1485.18	6160
Exercise efficiency (*N*=639)	− 2.25	0.23	0.07	0.63	0.92
Exam points (*N*=552)	18	85	79.82	15.71	100

### Were all types of smart content helpful?

We conducted multiple regression analysis to estimate the potential impact of using the practice system on four performance measures that we mentioned in the “Performance measures” section. To control for any effect of a student’s prior knowledge, we included the pretest score as an additional factor in the regressions. For the regressions that were fitted for learning gains, we could not directly use the pretest score as a control variable, because learning gain was derived from the pretest score itself. The analysis has two parts.

First, we examine the role of having entered the system (about the half of students did not use the practice system at all) on each performance measure. For these, we create the dummy variable *has-activity* indicating if the student has at least one content activity viewed. Regressions were run building two models. The first model only includes pretest as predictor. The second model adds the *has-activity* dummy variable which takes value 1 if the student viewed at least one activity, and 0 otherwise. The value 0 served as the reference group. This model allows us to see the additional contribution of *has-activity* to explain the variance of the dependent variable. Results of the second model are reported in Table 6, showing that all models are significant and in all cases both pretest and has-activity are significant predictors (for learning gain, there was only one model). Table 6 also shows the standardized coefficient values (in parentheses) which shows that *has-activity* has an even stronger impact than pretest. These results clearly show that students who entered the system performed higher than their peers who did not use the practice system, regardless of their level of prior knowledge.

**Table 6:** The predictive influence of attempting an activity (*has-activity*) in the system, controlling for the effect of pretest

	Learning gain	Exercise points	Exercise efficiency	Exam points
Model *R*^2^	.023**	.140***	.151***	.092***
Pretest coef. (stand.)	–	876 (.125)**	.61 (.207)***	15.886 (.209)***
Has-activity coef. (stand.)	.090 (.153)**	1022 (.344)***	.394 (.315)***	6.631 (.205)***

Each column represents the dependent variable of the model. Significance is marked with *** *p*<.001, ** *p*<.01

To further investigate whether each type of content is related to performance increase, we fitted another series of multiple regression models using the data of the students who entered the system. Regressions models were run as in the previous series of regressions, but now, only the students that had at least done an activity (view an example, solve a problem) in the practice system were considered. Table 7 shows the results of the regression analyses. The estimates for the influence of each smart content type on the learning gain, exercise points, effectiveness score, and exam points are shown in the first, second, third, and fourth columns of this table, respectively. Overall, each individual content type in Python Grids was found to be a significant factor for the increase in learning gain. Action-focused content (semantic and Parson’s problems) showed stronger relationship than the instructional content (annotated and animated examples). For example, each semantic problem or Parson’s problem solved accounts for 0.6% of the learning gain (range between 0 and 1). Each instructional content (examples) accounted for roughly the half of these values. The positive effect of problems decreases for exercise points and disappears for other performance measures. No relationship was found between examples and other performance measures.

**Table 7:** Regression estimates (standardized coefficients) for the impact of each type of smart content on students performance

	Learning gain		Exercise points		Exercise efficiency		Exam points	
	R2	Coef (stand.)	R2	Coef (stand.)	R2	Coef (stand.)	R2	Coef (stand.)
Semantic prob.	.050***	.006 (.224)***	.052*	13.978 (.127)*	.097 ^*#*^	.005 (.100) ^*#*^	.055	.117 (.076)
Parson’s prob.	.047**	.006 (.217)**	.051*	13.854 (.126)*	.097 ^*#*^	.005 (.102) ^*#*^	.054	.117 (.076)
Annotated examples	.034**	.003 (.184)**	.043	5.425 (.086)	.090	.002 (.055)	.050	.035 (.040)
Animated examples	.022*	.003 (.149)*	.038	4.174 (.048)	.088	.001 (.025)	.049	− .010 (− .008)

Significance is marked with *** *p*<.001, ** *p*<.01, * *p*<.05, ^*#*^*p*<.1

Furthermore, a regression analysis on learning gain was conducted adding each type of content following a stepwise approach. The goal is to see if all types of content are showing one or more effects. Only the number of parameterized semantic problems entered the regression model ($\phantom {\dot {i}\!}\beta _{{\text {parameterized}_{\text {p}}\text {rob}}}=.006$, $\phantom {\dot {i}\!}p_{{\text {parameterized}_{\text {p}}\text {rob}}}<.001$), and other types of content did not add predictive power ($\phantom {\dot {i}\!}p_{{\text {parson}_{\text {p}}\text {rob}}}=.482$, *p*_examples_=.669, *p*_animated_=.856). Among all content types, the amount of practice on parameterized semantic problems yielded stronger relationship with the learning gain.

These results demonstrate that using the practice system is positively related with performance and that the effect is stronger for self-assessment content (semantic and Parson’s problems). However, it is still not evident whether it is just the *total volume* or the *diversity* of the accessed content that matters. To further investigate the extent to which higher performance is influenced by accessing multiple types of smart content in the system, the rest of this section examines whether students used multiple content types (the “Were students using multiple types of smart content?” section) and also how student performance was affected by the usage of multiple types of smart content (“The value of practicing with multiple types of smart content” section).

### Were students using multiple types of smart content?

This subsection examines to what extent students practiced with multiple, rather than single types of smart content. To answer this question, we grouped the usage sessions of all students based on diversity of content types in those sessions. A session was defined as a sequence of consecutive activities of students where each activity occurred within 90 min of its preceding activity.

**Fig. 7:**
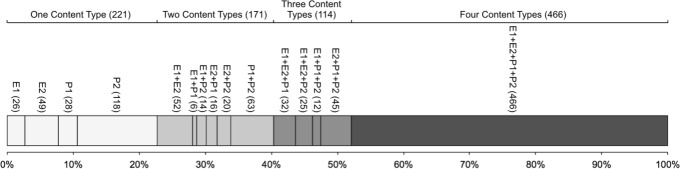
Distribution of session types for a total of 972 sessions in Python Grids. The session type is defined based on the types of smart content that students used in a session. In each session type, *E1*, *E2*, *P1*, and *P2* represent annotated examples, animated examples, Parson problems, and parameterized problems, respectively. The numbers in parentheses show the frequency of session type

In total, we identified 1612 sessions for 485 students who logged into the system. In 640 (39.7%) of these sessions, students did not attempt any content and likely logged in just to examine their progress. In the remaining 972 (60.3%) sessions, students used at least one content type. Figure 7 shows the distribution of session types by visualizing the ratio of various combinations of content types that students used during their practice sessions. As the figure shows, the majority of sessions were dedicated to practicing with multiple content types. There were 221 (22.7%) sessions with a single content type, 171 (17.6%) sessions with two content types, 114 (11.7%) sessions with three content types, and 466 (47.9%) sessions with all four content types.

Based on this observation, in less than 1/4 of the sessions, which included interactions with learning activities, students used a single smart content type, while in more than 3/4 of those sessions, students explored at least two types of content. And, more importantly, the number of sessions in which students interacted with all types of content is about twice the number of sessions where only one content type was used. Further comparison of single-content and multi-content sessions indicated that multi-content sessions (*M*=1.55,SE=0.19) were significantly more frequent than the single-content sessions (*M*=0.46,SE=0.1), Wilcoxon Signed-Ranks Z-statistic = 48,124, *p*<0.001. These results demonstrate that students had a significant tendency to work with multiple kinds of content within the same session. This provides good evidence in favor of integrating multiple kinds of smart content in one system, as supported by Python Grids.

Table 8 presents the relationships between the performance of students who logged into the system and the number of sessions where students used no content (0), one content type (1), at least one content type (1+), and two or more content types (2+). As the table shows, the number of sessions in each case is not correlated with the prior knowledge of students that is measured by the pretest score. This is an important finding, as it rules out the issue of experience-based selection bias. We also observed that there is no correlation between the number of sessions where none or one smart content type was used and any of the performance measures. On the contrary, there was a significant positive correlation between the number of sessions with multiple content types (1+, 2+) and all of the performance measures.

**Table 8:** Spearman correlation of student performance and number of sessions where students worked with zero (0), one (1), at least one (1+), and two or more (2+) content types

	Number of content types used in session			
Performance measure	0	1	1+	2+
Pretest score (*N*=471)	0.03	0.04	0.05	0.03
Learning gain (*N*=320)	− 0.01	0.05	0.15 ^∗∗^	0.19 ^∗∗∗^
Exercise points (*N*=485)	− 0.02	0.08	0.23 ^∗∗∗^	0.24 ^∗∗∗^
Exercise efficiency (*N*=485)	− 0.04	0.06	0.20 ^∗∗∗^	0.21 ^∗∗∗^
Exam points (*N*=439)	− 0.09	0.03	0.21 ^∗∗∗^	0.22 ^∗∗∗^

^∗∗^*p*<.01; ^∗∗∗^*p*<.001

**Fig. 8:**
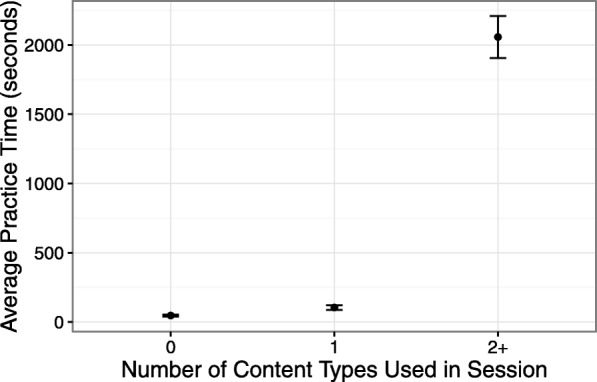
The average time (in seconds) of students’ practice in sessions where students worked with zero (0), one (1), and two or more (2+) content types. Error bars indicate standard error of the mean

We also looked into students’ practice time in sessions where students accessed different numbers of smart content types. Figure 8 shows the average amount of time (in seconds) that students spent in sessions where they practiced with zero (0), one (1), and two or more (2+) content types. As the figure shows, on average, the amount of time students spent in sessions where no content types were accessed was very short, usually less than a minute (*M*=46.63 s,SE=7.59). The average length of sessions where student practiced with only a single type of content was just a bit higher at 1.7 min (*M*=104.74 s,SE=17.13). On the other hand, the amount of time that students spent on sessions where they practiced with multiple types of content (2+) was much higher, about 34 min on average (*M*=2057.94 s,SE=152.23). Apparently, working with multiple content types was considerably more engaging, as students kept practicing about 20 times longer than in the sessions where they worked with only a single content type. This is another evidence that points to the importance of integrating multiple types of smart content within the practice systems.

Overall, our results highlight that students appreciated and benefited from the presence of multiple content types in Python Grids. On the one hand, they clearly preferred practicing with diverse content types. On the other hand, practicing with multiple smart content types was associated with higher course performance for students, regardless of their prior levels of knowledge.

### The value of practicing with multiple types of smart content

So far, we found that students, on average, had more sessions that included practicing with multiple types of smart content. While this clearly tells us about student preferences, it does not confirm an educational value of practicing with multiple types of content. In this section, we attempt to discover patterns of student work with practice content of different types and, ultimately, discover the impact of different practice patterns on student learning and performance. In the following, we look at two kinds of practice patterns with smart content. The “Value of practice with different combinations of smart content types” section attempts to discover and examine *type-related* patterns, i.e., how much students practiced with different types of smart content. The “Value of practice in different periods during the course” section examines *time-related* patterns, i.e., how early or late in the course students practiced with smart content. We show how each discovered practice pattern could impact student learning and performance in the course.

#### Value of practice with different combinations of smart content types

In the previous sections, we examined the educational value and appeal of different types of smart content considered in isolation. However, it could be a specific combination of content types, not individual types, that made students most engaged and/or efficient. In this section, we attempted to discover the most typical patterns of practice with different types of smart content. A content use pattern of a specific student can be represented as a vector of length 4 where each position represents how many times each content type (semantic problems, Parson problems, annotated examples, and animated examples) over the course duration. Since the number of possible content combination is too large (especially when distinguishing different usage balances within each pair of content types), the best way to find the most typical usage patterns is to cluster students based on their content use patterns. We conducted hierarchical clustering with the average-linkage method^6^ and cosine similarity as a measure of proximity between student usage of multiple content types. All students in the course (*N*=675) were clustered into four main groups, based on their practice styles (activities): one large group with no usage of the system (cluster *inactive*), two small groups with usage of a single content type (cluster *animations*, cluster *problems*), and one large group with usage of multiple types of content (cluster *mix*). Table 9 shows the usage of each type of smart content across these groups in Part (a).

**Table 9:** Summary of smart content usage and student performance across groups with different practice styles

	Cluster inactive (*N* = 339)	Cluster mix (*N* = 296)	Cluster animations (*N* = 25)	Cluster problems (*N*=15)
(a) Content usage	Mean (SE)	Mean (SE)	Mean (SE)	Mean (SE)
Semantic probs.	0 (0)	11.9 (0.56)	2 (0.74)	7 (2.52)
Parson probs.	0 (0)	11.59 (0.56)	2.6 (0.79)	0.4 (0.34)
Annotated ex.	0 (0)	20.61 (0.96)	1.72 (0.54)	0.87 (0.73)
Animated ex.	0 (0)	14.3 (0.71)	11.2 (2.02)	0.4 (0.4)
(b) Performance measure				
Pretest score	0.17 (0.01)	0.22 (0.01)	0.2 (0.05)	0.18 (0.06)
Learning gain	0.46 (0.02)	0.57 (0.02)	0.37 (0.06)	0.47 (0.08)
Exercise points	4110.74 (97.49)	5369.01 (61.14)	5045.88 (220.11)	5162.8 (328.61)
Exercise efficiency	− 0.21 (0.04)	0.28 (0.03)	0.12 (0.1)	0.19 (0.15)
Exam points	76.05 (1)	83.49 (0.83)	75.64 (4.36)	80.93 (4.19)
Practice time (minutes)	0 (0)	60.91 (5.12)	12.39 (3.13)	15.34 (6.54)

Cluster *inactive* consisted of 339 students that did not practice with the system, meaning that they never logged in, or that they logged in but did not access any content materials. Cluster *mix* was also a large group, which consisted of 296 students who worked on all types of smart content in a balanced way and had an average of more than 10 attempts on each type. Cluster *animations* consisted of 25 students who mainly practiced with animated examples: the average attempts on animated examples was about 11, while some content types had fewer than 3 attempts. Cluster *problems* was the smallest group; it consisted of only 15 students who used the system very little. Their practice was mostly with semantic problems, with an average of 7 attempts.

Table 9 shows the mean and standard error of performance measures across these four groups, in Part (b). As the table shows, cluster *mix*, which consisted of students practicing with multiple types of content, achieved higher performance in terms of learning gain, exercise points, exercise efficiency, and exam points. The ANOVA analysis showed significant main effects for clusters (practice style) on each of the performance measures^7^.

*Pretest score*. There was no significant difference among the pretest scores of clusters *mix*, *animations*, and *problems* who used the system. All of these groups had higher pretest scores, as compared to cluster *inactive*, who did not use the system. However, this difference was significant only for cluster *mix* and cluster *inactive* (by Tukey’s post hoc test at *p*=.025).

*Learning gain.* Tukey’s post hoc test showed a significant difference on the learning gain between cluster *mix*, who worked on multiple types of content, cluster *inactive*, who did not use the system (*p*=.001), and cluster *animations*, who used mostly animated examples (*p*=.014), with cluster *mix* obtaining higher learning gains than either clusters *inactive* or *animations*. There was no significant difference between the other groups.

*Exercise points and efficiency.* Tukey’s post hoc test indicated that all groups who practiced with the system obtained higher exercise points and efficiency than cluster *inactive*. More specifically, cluster *inactive* achieved fewer exercise points than cluster *mix* who practiced with multiple types of content (*p*<.001), and cluster *animations* (*p*=.013) and cluster *problems* (*p*=0.036) who used smart content but mostly worked with single content type. Similarly, exercise efficiency was significantly lower in cluster *inactive* than cluster *mix* (*p*<.001) and cluster *animations* (*p*=.045). Cluster *inactive* had also lower efficiency than cluster *problems*, but the difference reached only marginal significance (*p*=.067). The difference between the exercise points and efficiency was not significant among the groups who used the system though.

*Exam points.* Tukey’s post hoc test demonstrated a significant difference on the exam points between cluster *mix*, who worked on multiple types of content, and both clusters *inactive* (*p*<.001) and *animations* (*p*=.065), with cluster *mix* obtaining higher exam points than clusters *inactive* and *animations*. No significant difference was found between the other groups.

*Practice time.* The average time (in minutes) that students practiced with smart contents is shown in the bottom row of Table 9. On an average, students in cluster *mix* who worked with multiple types of content also spent more time practicing with the system than the students in the other clusters. The difference between the amounts of practice time was statistically significant when cluster *mix* was compared to cluster *inactive* (*p*<.0001), cluster *animations* (*p*<.001), and cluster *problems* (*p*=.018).

The above results point to a positive impact in practicing with smart content, as well as the style of practicing, on students’ performance. Although all practice styles that included work with single or multiple types of content correlated with good student performance on the course, mandatory exercises, positive correlation with learning gains, and increased exam scores were only observed for the practice style that included work on multiple types of content. This improved performance could be explained by the greater amount of time that students spent practicing with multiple types of content. This observation, in fact, highlights the importance of practicing with different types of content for learning programming—those students who work with multiple types of content tend to spend more time practicing and thus tend to achieve a better performance.

#### Value of practice in different periods during the course

**Fig. 9:**
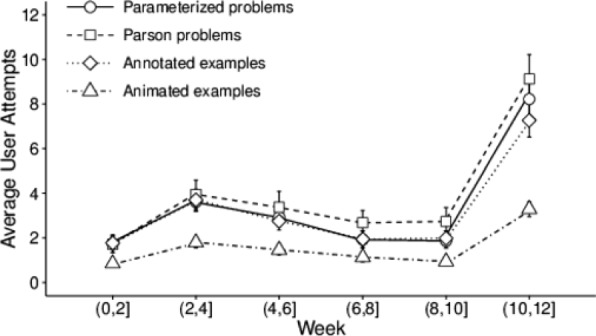
Average usage of smart content types for students in clusters *mix*, *animations*, and *problems* (*N*=336). The *y*-axis shows the average of students’ attempts on the smart content types, and the *x*-axis shows 2-week intervals of the course. Each point in the above plots represents the average of student attempts during the corresponding weeks in the course. Error bars represent standard error from the mean

The previous section examined how student practice was distributed over the *type* of practice content. Another dimension to examine is how this practice was distributed over *time*. First, we considered all students who used the system (clusters *mix*, *animations*, *problems*) and examined how much they practiced with different types of content during different weeks within the course. Figure 9 shows the average attempts on each content type over the course weeks for active students (all but cluster *inactive*). The data shows that on average, students consistently worked with multiple types of content during the course, with the exception of exam preparation weeks (week 10 to week 12) when the amount of work with all content types was much larger than average, especially for semantic problems, Parson problems, and annotated examples.

To investigate whether students differ by their distribution of practice over time, we clustered all students based on their ratio of work with each content type both before and during the exam preparation time (i.e., 1 week before the exam). Table 10 shows the three clusters that we obtained: The first cluster (*N*=339) includes students who did not use practice content, and we refer to them as cluster *inactive* (this includes the same students as cluster *inactive* in the previous clustering); cluster *exam* (*N*=118) includes students who had very few attempts during lecture weeks and used the system mostly for exam preparation; and cluster *regular* (*N*=218) represents students who used the system mostly during the lectures and who had only a relatively small fraction of attempts during the exam preparation time. Thus, these students had a more regular engagement with the Python Grids system during the term.

**Table 10:** Summary of smart content usage across different clusters when system usage is split into two parts: usage during course lectures, namely regular practice, and usage during exam preparation

	Regular Practice			Exam preparation		
(a) Content usage	Cluster inactive (*N*=339)	Cluster exam (*N*=118)	Cluster regular (*N*=218)	Cluster inactive (*N*=339)	Cluster exam (*N*=118)	Cluster regular (*N*=218)
Semantic probs. /|*P*1|	0 (0)	0.03 (0.01)	0.24 (0.01)	0 (0)	0.32 (0.03)	0.03 (0)
Parson probs. /|*P*2|	0 (0)	0.04 (0.01)	0.31 (0.02)	0 (0)	0.38 (0.03)	0.03 (0.01)
Annotated Ex. /|*E*1|	0 (0)	0.04 (0.01)	0.28 (0.02)	0 (0)	0.28 (0.03)	0.03 (0.01)
Animated Ex. /|*E*2|	0 (0)	0.04 (0.01)	0.31 (0.02)	0 (0)	0.3 (0.03)	0.03 (0.01)
Practice time (minutes)	0 (0)	11.06 (5.45)	44.81 (3.78)	0 (0)	69.68 (10.12)	4.96 (0.95)

|*P*1|, |*P*2|, |*E*1|, and |*E*2| indicate the number of semantic problems, Parson problems, annotated examples, and animated examples, respectively

Usage is expressed as mean and standard error for the mean (in parentheses)

As the bottom row of Table 10 shows, students in the cluster *regular* spent, on average, significantly more time practicing with the system during the course lecturing period than students in the cluster *exam* (*p*<.0001) and cluster *inactive* (*p*<.0001). Students in cluster *exam* practiced with the system during the course lecture period as well, and the average time of their practice was significantly greater than zero (i.e., the practice time of students in cluster *inactive*), yet their average practice time was about one quarter of the time that students spent on practice in cluster *regular*. The practice time of students in each cluster changes when we look at the usage during exam preparation. In particular, the average practice time of students in cluster *regular* did not differ significantly from zero (i.e., the practice time of students in cluster *inactive*), while students in cluster *exam* spent significantly more time than students in both clusters *inactive* (*p*<.0001) and *regular* (*p*<.0001).

To compare the performance of these clusters, we looked at student learning and exam points. We ignored student performance on exercises, because students in cluster *exam* had too little practice with the system during the course lecture period, and as a result, their performance on exercises could be hardly attributed to the system usage. Table 11 shows performance of the three clusters in terms of pretest scores, learning gain, and exam points.

**Table 11:** Summary of student performance for clusters obtained after splitting usage into usage during course lectures and usage during exam preparation

Performance measure	Cluster inactive (*N*=339)	Cluster exam (*N*=118)	Cluster regular (*N*=218)
Pretest score	0.17 (0.01)	0.17 (0.01)	0.24 (0.02)
Learning gain	0.46 (0.02)	0.53 (0.03)	0.56 (0.02)
Exam points	76.05 (1)	79.96 (1.37)	84.38 (1.03)

Values are expressed as mean and standard error for the mean (in parentheses)

The ANOVA main effects showed a significant impact of each cluster on the performance measures^8^. Significant effects were further examined with Tukey’s post hoc test. There was no significant difference in pretest scores between clusters *inactive* and *exam*; however, cluster *regular* had significantly higher pretest scores as compared to both clusters *inactive* (*p*<.001) and *exam* (*p*=.005). This may have been the reason that students in cluster *regular* had better practice habits. In terms of learning gain, both students in clusters *exam* and *regular* who used the system either during the lectures or exam preparation performed comparably well (i.e., no significant difference) and had higher learning gain, as compared to those in cluster *inactive* who did not use the system. Yet, only cluster *regular* reached a significant difference with cluster *inactive* (*p*=.005). In terms of exam points, cluster *regular* had a significant difference with cluster *inactive* (*p*<.0001), while the difference between cluster *inactive* and cluster *exam* was only marginally significant (*p*=.057). Additionally, there was a significant difference between clusters *regular* and *exam*, with cluster *regular* having significantly higher exam points than cluster *inactive* (*p*=.033).

In addition, to make sure that a cluster’s behavior was not influenced by a group of students who dropped the course, we repeated clustering after discarding the data of 95 students who dropped the course. These students did not obtain the minimum number exercise points from the first eight rounds of mandatory exercises that were required to pass the course. The majority of students (84 of 95) who dropped the course belonged to cluster *inactive* who never used the system at all; one student was in cluster *exam,* and the remaining 10 were in cluster *regular*. The updated clustering results showed almost no change in the behavior and performance of clusters *inactive*, *exam*, and *regular*.

The above results confirm that students who worked with practice content performed better in respect to all performance measures than those who did not practice with the system at all. Moreover, we found that among those who practiced with the system, students who regularly practiced with smart content achieved higher learning outcomes than students who used practice content mostly for exam preparation. Both styles of practice led to comparable learning gains. When considering learning gain, which was assessed on exactly the same type of problems that students practiced, as an indicator of near transfer, we can say that both types of practice will lead to comparable performance on near transfer tests. However, only students with regular practice were able to perform better on exams, which could be considered to be a far transfer test.

We conjecture that the better performance of students in cluster *regular* as compared with students in cluster *exam* stems from the fact that students in cluster *regular* spent more time practicing with the smart content during the course lecture period. Interestingly, when we looked at the total time that students in clusters *exam* and *regular* spent on practicing during the whole semester (i.e., the amount of practice time during the course lectures and exam preparation), we found that the average practice time was significantly higher for students in cluster *exam*, who practiced more during exam preparation (*M*=72.1 min, SE=11 min), than those in cluster *regular*, who practiced regularly during the course lectures (*M*=46.16 min, SE=3.75 min) (*p*<.001). This implies that it was the regular practice that led to better performance in cluster *regular* and that even the extra time spent during exam preparation in cluster *exam* was not able to beat the positive influence that regular practice had on student performance. Finally, it should be noted that the observed effects could not be attributed solely to having a high pretest score. As our analyses showed, the work in the practice system explained the performance even after considering the pretest.

### The impact of student motivation on using multiple types of smart content

#### Data collection and pre-processing

The motivation questionnaire was answered by 629 students (141 females) at the beginning of the term (initial) and 440 students (107 females, 16 undeclared) at the end of the term (final). Informed consent was collected from all students who answered this questionnaire. We performed reliability and factor analyses to verify that the questionnaire is a consistent and reliable measure of different motivational factors. After these analyses, we discarded mastery avoidance because of its low reliability and performance avoidance because it was not distinguished from performance approach. Based on these results, we decided to go forward with all three learning activation factors, which are fascination (F), competency beliefs (CB), and values (V), but we decided to proceed with only two factors of the achievement-goal orientation instrument, which are mastery approach (MAp) and performance approach (PAp).

For both *initial* (beginning of the term) and *final* (end of the term) questionnaire answers, the scores of each of the motivational factors were computed by averaging the scores of the associated items in the questionnaire. Since these motivational factors changed over the term (there is a difference between the initial and the final measure), we further computed the average between the initial and final measures (*N*=424,107 females) and considered this to be a representation of the middle state of the motivational profiles of the students. Table 12 shows the basic statistics of the motivational factors. Since the factors have different scales, and to facilitate the interpretation of the scores, we normalized the scores to the range of 0 (lower) to 1 (higher).

**Table 12:** Mean and standard deviation of the motivational factors, measured at initial (start of the term) and final (end of the term)

	Initial (*N*=629)		Average (middle) (N=424)		Final (*N*=440)	
	Mean	SD	Mean	SD	Mean	SD
F	0.565	0.162	0.566	0.160	0.577	0.196
CB	0.502	0.221	0.574	0.173	0.658	0.196
V	0.700	0.167	0.685	0.156	0.684	0.181
MAp	0.692	0.171	0.666	0.158	0.643	0.200
PAp	0.573	0.239	0.577	0.198	0.568	0.225

#### Motivation differences among students using different types of content

To explore the relation of the motivation and the usage of different types of content, we ran an ANOVA test on each of the motivational factor scores (F, CB, V, MAp, PAp) among the clusters *inactive*, *mix*, *animations*, and *problems*. Table 13 summarizes the result of the ANOVA test among the four clusters.

**Table 13:** Results of ANOVA and post hoc multiple comparisons (with Bonferroni correction) on motivational factors among four clusters (“Value of practice with different combinations of smart content types” section)

	*M* (SD)				*p* value	Mult. compar. (*p*value)	
	Inactive	Mix	Animations	Problems	(partial *η*^2^)	Inactive, mix	Mix, animations
F	.531 (.141)	.590 (.166)	.527 (.133)	.617 (.204)	.001 (.036)	.002	
CB	.541 (.168)	.600 (.174)	.502 (.140)	.627 (.183)	.001 (.038)	.005	.062
V	.666 (.146)	.703 (.163)	.639 (.127)	.658 (.158)	.056 (.018)		
MAp	.626 (.157)	.697 (.156)	.628 (.099)	.646 (.169)	< .001 (.049)	< .001	
PAp	.549 (.184)	.600 (.211)	.540 (.127)	.566 (.188)	.061 (.017)	.064	

Only significant and marginally significant differences are reported

There are significant differences on fascination, competency beliefs, and mastery approach orientations, and marginally significant differences on values and performance approach orientations. Multiple comparisons reveal that most of the difference lies between clusters *inactive* and *mix*. In general, students in cluster *mix* exhibit higher levels of motivation (in all factors) than those in cluster *inactive*, and the highest difference is in the mastery approach orientation. This suggests that students who use the system considerably are distinguished from those who did not use the system at all by their intrinsic motivation to learn and not just a desire to perform well on tests.

The data also show a marginally significant difference in competency beliefs between clusters *mix* and *animations*. Students in cluster *mix* had marginally higher competency beliefs than students in cluster *animations*. Interestingly, we found no significant difference among pretest scores between students in clusters *mix* and *animations*. This may suggest that students in cluster *animations* were not weaker than students in cluster *mix* but that they believed less in their own competency and, as a result, were more eager to work more with instructional content (in our case, animated examples) rather than practice with self-assessment content. This is intriguing, although it is not conclusive, because of the limited power of the analysis (cluster *animations*, *N*=22).

#### Motivation differences among students practicing in different periods during the course

Similarly to the previous section, we performed an ANOVA analysis on all motivational factors among clusters *inactive*, *exam*, and *regular* that represented different regularity on the usage of the system within the term defined in the “Value of practice in different periods during the course” section. Results are shown in Table 14.

**Table 14:** Results of ANOVA and post hoc multiple comparisons on motivational factors among three clusters (the “Value of practice in different periods during the course” section)

	*M* (SD)			*p*value	Mult. comparisons (*p*value)		
	Inactive	Exam	Regular	partial *η*^2^	Inactive, exam	Inactive, regular	Exam, regular
F	.531 (.141)	.545 (.162)	.613 (.166)	< .001 (.055)		< .001	
CB	.541 (.168)	.559 (.180)	.614 (.167)	< .001 (.036)		< .001	
V	.666 (.146)	.665 (.150)	.714 (.164)	.007 (.023)		.014	.039
MAp	.626 (.157)	.669 (.156)	.703 (.153)	< .001 (.046)	.099	< .001	
PAp	.549 (.184)	.580 (.191)	.604 (.211)	.040 (.015)		.034	

Only significant and marginally significant differences are reported

Overall, there are significant differences on all motivational factors among the clusters. Most essentially, cluster *regular* exhibits higher levels of motivation than cluster *inactive*. It is interesting that there are no significant differences between clusters *inactive* and *exam*, which suggests that students who react to exam pressure are not *motivationally* different than those who did not use the system at all. Another interesting observation is the difference in values among clusters *exam* and *regular* (the only significant difference between these clusters). It reveals that students who use the system regularly only differ from those focused on exam preparation in declaring higher values towards learning programming. It seems that although values do not strongly distinguish students who use the system or not (it was not significant in the previous analyses in the “Motivation differences among students using different types of content” section), they do help to explain a regular practice over the course duration that was shown as a positive factor affecting exam performance.

### Profiles of students who did not use the system

Roughly half of the students did not use the practice system at all (*N*=339). Note that the very idea of a “practice” system is that it offers additional practice for students who are interested to improve or expand their knowledge. The use of the system was not a required part of the course, although the instructor encouraged students to use it citing its educational value and offered small credit for using it. The students who never used the system could skip it for a number of reasons. Some might consider that their knowledge is strong and they do not need additional practice. Some might have no extra time. Some might not be striving to do well in the course. Our data, however, offers an opportunity to take a deeper look at this group of students. This subsection attempts to uncover the reasons for *inactive* students not to use Python Grids by comparing it with the rest of the class in different aspects.

A natural hypothesis to assess is whether *inactive* students have lower engagement with the course in all aspects, not just the practice system. This hypothesis could be checked by comparing the completion of the course and the completion of the mandatory content (mandatory exercises that contributed to the overall course grade) between *inactive* students and the rest of the class. Our data shows that in the group who did not engage with the practice content (cluster *inactive*, *N*=339), 30% did not complete the course (i.e., did not take the required final exam). This proportion is considerably different for the groups of students who used practice content: 2% in cluster *exam* and 9% in cluster *regular*.

Similarly, 70% of students who did not use the practice content did not complete all mandatory content either. These proportions are only 44% and 42% in clusters *exam* and *regular*, respectively. Even when removing from consideration students who did not finish the course, students in *inactive* cluster were significantly more likely to leave mandatory content incomplete, *χ*^2^(1549)=23.134,*p*<.001.

The motivation analysis reported in the “Motivation differences among students using different types of content” and “Motivation differences among students practicing in different periods during the course” sections indicates that the most likely reason for the low levels of course engagement within cluster *inactive* is not just the lack of time, but the lack of motivation. As Tables 13 and 14 show, these students are consistently less motivated, showing less fascination and lower values for the subject, and are biased against mastery-oriented goals. Even when removing students who did not finish the course, motivational differences between clusters present the same pattern, i.e., among those who completed the course, the group of students who did not engage with the practice content were still consistently less motivated. A related reason could be a lack of knowledge, which could cause students to struggle in the course. As Table 9 shows, students in cluster *inactive* have lower pre-test scores and ended the course with a considerably lower knowledge gain and lower exam points than the majority of students who use the system (cluster *mix*).

## Summary and conclusions

### Contributions and outcomes

Here, we summarize our main contributions and findings.

- We developed an open architecture for integrating several different types of smart learning content. Using this architecture, we built Python Grids, a practice system for learning Python that used four types of learning content hosted at servers both in the USA and in Finland.
- The Python Grids was deployed in a large CS1 programming course and was used to carry out a large-scale study to investigate the value of using multiple content types in this important context.
- We found that students appreciated the presence of multiple content types in Python Grids and preferred practicing with diverse content types; there were more sessions that student accessed multiple content types than only one type of content.
- Our results indicate that every type of content matters and is important to student learning. Practicing with multiple smart content types was associated with higher course performance for students, regardless of their prior levels of knowledge.
- Moreover, although all practice styles that included work with single or multiple types of content helped students to perform better on the course’s mandatory exercises (as compared with those who did not access any practice content), a positive effect on learning gain and exam score was only observed for practice styles that included work with multiple types of practice content.
- Students who worked with practice content achieved superior performance than those who did not practice with the system at all. Among those who practiced with the system, students who regularly practiced with non-mandatory practice content achieved higher learning outcomes than students who used practice content mostly for exam preparation. Only students who engaged in regular practice were able to perform better on the final exam.
- Students who used the system had higher levels of motivation than those who did not use the system, especially the students who used the system regularly. Motivational differences might explain the different types of content usage, and we observed that the group who preferred to solve problems were characterized by a higher belief in their own competencies. The motivational profile also showed that students who used the system when they were reacting to the pressure of the exam gave less value to the importance of the subject (values), as compared to students who used the system regularly.
- On the other hand, students who did not use Python Grids were consistently less motivated, and this difference is stronger in intrinsic motivational traits. This group of students included a much higher proportion of students who did not complete the course and did not complete mandatory content, as compared with the group of students who actively used the practice system. Even within those who finished the course, the students who did not use the Python Grids system were consistently less motivated.

### Discussion

We argue that there are several reasons to provide students with various types of smart content. From a practical point of view, one of the key aspects of improving learning results is engaging students through motivating content and assignments. We consider content diversity as a positive asset. Students are likely to have different preferences on which kind of content is engaging and helpful for their learning. These preferences can be related to several things, like student background, previous knowledge, or studying practices and skills. These considerations are also supported by the variation theory (Mun Ling and Marton 2011), which suggests that to achieve deep learning results, students should discern different dimensions of variation on the concepts they are learning. Multiple types of content allow them to explore different aspects of content and, thus, help them to achieve this goal.

From a research point of view, the use of multiple types of learning content is important to better understand the process of human learning. Humans naturally perform different kinds of activities in their learning process. The use of multiple learning content types allows not only better support these different activities, but also capture data about a larger part of the learning process, i.e., when a student solving programming problem using smart content rather than a compiler or paper and pencil, we can capture the problem solving process in details. When a student works with program examples using interactive smart tools rather than a textbook, we can capture and understand how humans learn from examples. In this sense, the more kinds of smart content is offered, the larger is the fraction of learning that we can capture, the better is our ability to study human learning. In this aspect, educational systems with multiple kinds of smart learning content could enable researchers to move from a mere evaluation of isolated tools to studying the whole learning process.

While these arguments provide good support for using multiple types of smart content, there can be major technical challenges in implementing this goal. Integrating static content, like text, images, and videos^9^ in learning management systems and portals is straightforward. However, integrating smart content is not, because a smart content item is not a static file, but rather a process that should be executed either in a browser, on a server, or both. To support its functionality and store the details of student work, such content usually needs to communicate with other components of the learning process, such as learning portals for accessing student information and storing results (e.g., student IDs, grades, or exercise points), as well as with learning record storage and student modeling component to log data about student interaction and progress. The complexity of supporting student work with smart content usually calls for encapsulating each kind of smart content in its own login-based system. In turn, from student’s point of view, the parallel usage of several types of content tends to be prohibitive, even if open-source content is provided.

Following the working group report (Brusilovsky et al. 2014), we argue that practical use of multiple types of smart content requires a system architecture that supports flexible re-use and single-point access to a variety of smart content. Python Grids, built on such an architecture, demonstrates the feasibility of integrating resources from various providers and providing diverse materials for students.

Our reported experience of using Python Grids in a large-scale course provides several arguments in favor of learning with multiple kinds of smart content. Our results also corroborate and extend results of previous research on using ebooks with smart content. A majority of students who used voluntary practice content were engaged with multiple types of content. This is in line with large-scale studies on the use of “smart ebooks” by CS teachers and students reported in Ericson et al. (2015a); Ericson et al. (2016). The study Alvarado et al. (2012) showed that the dominated majority of students exhibited very low usage level of smart widgets in an ebook, although all students showed at least some activity. Our data showed that a significant share of students did not use the practice system at all. However, in our study, all smart content was located in an external non-mandatory practice system Mastery Grids, which was easier to ignore, while in the study by Alvarado et al. (2012), all material was integrated into the ebook along with the primary learning content.

In our study, using multiple types of content directly correlated with students’ overall success. Furthermore, those students who used multiple types of content regularly performed better than those who used such content only when preparing for the exam. This can be explained by the increased working time of the content, but this is exactly where fluent integration of smart content aims at: easier usage promotes more engagement and as a consequence better learning results. Our results also implied that regular usage leads to better performance, which fits well with the previous conclusion. Better results of regular users could also be explained by better learning habits, but also then easy usage through integration is a central asset. Compared with the results by Alvarado et al. (2012), which showed better learning results only in the context of using CodeLens visualizations of program execution, our results thus supports the hypothesis of the benefits of engaging students with multiple types of content.

The pedagogical side of using multiple types of content raises the issue of course requirements: should we enforce student work with different types of content or should using such content be strictly voluntary? In our research setting, all practice content available in the Python Grids system was non-mandatory, yet almost half of the students (of those who used the system) used all four types of Python Grids content and less than one-fourth focused on one type of content. As two content types (animated examples and Parson’s problems) were also used in the mandatory content, we investigated whether this had an effect on the use of voluntary content types. We found no evidence that students would ignore voluntary practice content of a specific type if they used this type of content in the mandatory exercises, i.e., students considered it to be worthwhile to continue working with many types of content.

On the other hand, when smart content is offered in the form of non-mandatory practice, its usage could be too low, especially among less motivated students. Even for highly engaging and attractive content, it could be challenging to ensure that the students had a chance to try it. To address this problem in our study, we allocated some bonus points to prompt student work with voluntary practice content in Python Grids. However, the number of these bonus points was very small: only 3% of the total number of points. It is thus likely that the extra effort in using the content was almost solely due to intrinsic value of this content and other motivational factors rather than to the availability of bonus points. We believe that students who used the voluntary content considered practice example and problems to be both interesting and beneficial per se. This assumption is supported by the large group, roughly half of the students, who did not use the system at all even when they missed to get the bonus points. The same phenomenon was observed in our previous, unpublished study a year earlier, when only a minority of students accessed the voluntary practice content despite of bonus points^10^.

Given the results of our data analysis, we can argue that the ability to work with multiple types of smart content contributed to student motivation to work with the system. Our experience favored a policy of offering multiple kinds of smart content as non-mandatory *practice content*, possibly accompanied with a bonus, instead of setting it as obligatory. However, more research is needed to confirm this assumption.

Another pedagogical challenge of organizing student work with smart content is ensuring the regular use of such content. As our data shows, the regular use of smart content correlates with better performance and better course outcomes. The value of regular practice is also supported by modern research on spaced learning (Carpenter et al. 2012). While the spaced use of smart content released in a mandatory form could be enforced through deadlines, a non-mandatory use of practice content could easily lead to procrastination in starting to work with this content and delaying its use until exam preparation. The results of our earlier research indicate that personalized content guidance that recommend what content to use and when could partially address this problem (Hosseini et al. 2015); however, further research is necessary to establish best practices in using smart content in a non-mandatory form.

### Limitations

In this work, we focused on the technical integration of *smart* content of different types and from different providers, as well as on the advantages of offering such diverse content on student engagement and levels of learning. As an early exploration of this topic, our research and findings are subject to several limitations. First, we recognize that our results could be related to the four specific types of smart content that we used in our study, even though they had clearly different characteristics in terms of engaging students. More research with a broader variety of content is required to generalize our results. We are currently working on integrating and exploring other types of content that may have a different impact on engagement and learning, such as lecture videos, worked-out examples, and code construction problems.

Another limitation is related to the very nature of our non-mandatory practice content: it made our results subject to a *self-selection bias*. In particularly, we observed that students with higher prior knowledge (higher pretest) worked more regularly with the system than students with lower prior knowledge (lower pretest). This means that we cannot reliably separate the effect of regular usage from the effect of prior knowledge on student performance. To check to what extent the self-selection bias might have affected our results, we examined the correlation between pre-test and user activity. Our data suggests that there is a weak positive relationship between the students’ pre-test score and their performance in solving Parson’s problems (*ρ*=0.1,*p*<.01). This confirms the existence of the self-selection bias but at the same time shows that this bias explains only a fraction of overall engagement. While this data hints that the results reported in this paper are informative despite of the observed bias, further study is required to investigate how we can engage students with lower prior knowledge to use multiple types of content and then assess the value of practicing with multiple types of content on their performance.

The self-selection bias also makes it harder to reliably assess an impact of a specific kind of smart content or its combination on student learning. Since it was up to the student whether to practice with a specific kind of content, the potential benefits of specific content types were intermixed with a range of factors that cause the students to select this content (i.e., prior knowledge, learning preferences, use of other ways to practice, such as study groups, etc.). In order to fully evaluate the learning impact of smart content, a classroom study should be complemented with a carefully designed controlled study were comparable group of students will practice with specific types of content and without access to alternative ways to gain knowledge.

Similarly, the lack of a control group that used multiple types of contents without an advanced Mastery Grids interface makes it harder to isolate the value of using multiple types of content from the added value of an advanced progress-tracking interface. This limitation, however, is directly related to the technical side of the paper: without an integration platform, such as Python Grids, it would be exceptionally hard to study student works with multiple kinds of smart content due to a range of problems discussed in Brusilovsky et al. (2014). As different integration platforms become available, it will become possible to separately assess the value of each platform type, for example, centralized progress-based interface as in Python Grids vs. textbook-style interface as in Ericson et al. (2016) or Fouh et al. (2014).

A more specific limitation is the use of a self-reported amount of time as a part of the efficiency measure. It is well known that self-reported measures contain biases. In our case, there might be students who overestimated or underestimated the amount of time they spent on an exercise. To minimize this problem, we discarded the extreme self-reported values from our analysis; however, this does not guarantee a complete removal of bias.

### Future work

There are several directions in which the present research could be extended. To check and generalize our finding about the value of multiple types of smart content in a practice system on student engagement and learning, we plan to perform similar studies with other domains (such as Java and SQL) and with other types of content (such as worked-out examples and programming problems), and other audiences (i.e., students from different countries, at different course levels, or with different levels of expertise). The current infrastructure makes it easy to add new types of smart content and explore it in different combinations. We also want to broaden the set of collected data to better explain engagement differences with the practice content. For example, capturing subjective evaluations of perceived usefulness, usability, and satisfaction could bring in useful information to evaluate different features of the system and the support that different content provides to students, even at different times of the term.

Second, while our results imply that it would be worthwhile to encourage instructors of programming courses to include multiple types of smart contents in their course materials, some questions remain for further analysis. What is the best strategy that instructors should employ to increase the effect of learning from multiple types of content? Should the variety of smart content types be offered as voluntary practice content for the course? Should the instructors enforce the use of multiple types of content? Should they use a combined approach, as in the present study, that offers some of the content as mandatory and the rest as practice content? All these questions point to the need for further research that could provide a guideline for the practical use of multiple types of content in the classroom setting. A further dimension here is the considerably large group of students who never used the system. Understanding their motivation or lack of it would support designing better guidelines on how practice content should be integrated into courses.

Another direction for future work, which could affect the selection of effective strategies for use of multiple content types in classroom settings, relates to exploring the effect of various engagement strategies to encourage the use of practice contents. In particular, we plan to broadly examine the impact of open social student modeling. We have evidence from our prior work that social comparison increases user engagement to work with practice systems. Future studies should examine the role of social comparison features in encouraging the use of multiple types of practice content across diverse student populations, possibly covering a range of populations with subtle cultural and educational system differences. More broadly, in our future work, we would like to compare Python Grids, which offers a social comparison option, to other interfaces and systems for accessing multiple types of course materials.

## Footnotes

1 http://opendsa.org/.

2
https://plus.cs.hut.fi/o1/2018/

3 (https://js-parsons.github.io/).

4 http://acos.cs.hut.fi/.

5 http://adapt2.sis.pitt.edu/wiki/CUMULATE_protocol.

6 In the average-linkage method, the distance between two clusters is calculated by taking the average of all pair-wise distances between the members of the two clusters

7 Pretest score: *F*(3395)=2.7,*p*=.045; learning gain: *F*(3395)=6.6,*p*<.001; exercise points *F*(3671)=39.0,*p*<.001; exercise efficiency *F*(3671)=34.4,*p*<.001; exam points *F*(3565)=11.2,*p*<.001.

8 Pretest score: *F*(2396)=8.8,*p*<.001; learning gain: *F*(2396)=5.1,*p*<.01; exam points *F*(2566)=16.8,*p*<.0001.

9 We consider videos to be static content in the sense that the content does not change in response to student actions, like pausing and proceeding.

10 We speculate that this might be a cultural issue, because in Finland students in technical sciences are generally not very competitive - certificate grades have often less meaning for getting employed than work experience gained from internships and part time work during studying.
